# KLK5 Inactivation Reverses Cutaneous Hallmarks of Netherton Syndrome

**DOI:** 10.1371/journal.pgen.1005389

**Published:** 2015-09-21

**Authors:** Laetitia Furio, Georgios Pampalakis, Iacovos P. Michael, Andras Nagy, Georgia Sotiropoulou, Alain Hovnanian

**Affiliations:** 1 INSERM UMR 1163, Laboratory of Genetic Skin Diseases, Imagine Institute, Paris, France; 2 University Paris Descartes - Sorbonne Paris Cité, Paris, France; 3 Department of Pharmacy, School of Health Sciences, University of Patras, Rion-Patras, Greece; 4 Samuel Lunenfeld-Tanenbaum Research Institute, Mount Sinai Hospital, Toronto, Ontario, Canada; 5 Department of Genetics, Necker Hospital, Paris, France; University of California, San Diego, UNITED STATES

## Abstract

Netherton Syndrome (NS) is a rare and severe autosomal recessive skin disease which can be life-threatening in infants. The disease is characterized by extensive skin desquamation, inflammation, allergic manifestations and hair shaft defects. NS is caused by loss-of-function mutations in *SPINK5* encoding the LEKTI serine protease inhibitor. LEKTI deficiency results in unopposed activities of kallikrein-related peptidases (KLKs) and aberrantly increased proteolysis in the epidermis. *Spink5* ^*-/-*^ mice recapitulate the NS phenotype, display enhanced epidermal Klk5 and Klk7 protease activities and die within a few hours after birth because of a severe skin barrier defect. However the contribution of these various proteases in the physiopathology remains to be determined. In this study, we developed a new murine model in which *Klk5* and *Spink5* were both knocked out to assess whether Klk5 deletion is sufficient to reverse the NS phenotype in *Spink5* ^*-/-*^ mice. By repeated intercrossing between *Klk5* ^*-/-*^ mice with *Spink5* ^*-/-*^ mice, we generated *Spink5* ^*-/-*^ *Klk5* ^*-/-*^ animals. We showed that *Klk5* knock-out in Lekti-deficient newborn mice rescues neonatal lethality, reverses the severe skin barrier defect, restores epidermal structure and prevents skin inflammation. Specifically, using *in situ* zymography and specific protease substrates, we showed that *Klk5* knockout reduced epidermal proteolytic activity, particularly its downstream targets proteases KLK7, KLK14 and ELA2. By immunostaining, western blot, histology and electron microscopy analyses, we provide evidence that desmosomes and corneodesmosomes remain intact and that epidermal differentiation is restored in *Spink5* ^*-/-*^ *Klk5* ^*-/-*^. Quantitative RT-PCR analyses and immunostainings revealed absence of inflammation and allergy in *Spink5* ^*-/-*^ *Klk5* ^*-/-*^ skin. Notably, Il-1β, Il17A and Tslp levels were normalized. Our results provide *in vivo* evidence that KLK5 knockout is sufficient to reverse NS-like symptoms manifested in *Spink5* ^*-/-*^ skin. These findings illustrate the crucial role of protease regulation in skin homeostasis and inflammation, and establish KLK5 inhibition as a major therapeutic target for NS.

## Author Summary

Netherton Syndrome (NS) is a severe form of ichthyosis characterized by desquamation, inflammation and multiple allergies, which can be life-threatening in infants. NS is caused by loss-of-function mutations in *SPINK5* encoding the LEKTI serine protease inhibitor. Current treatment options for this orphan disease are non-curative, focusing on the management of skin infections and the reduction of itching and pain. We developed a new murine model in which *Klk5* and *Spink5* were both inactivated to assess whether *Klk5* loss is sufficient to reverse the NS phenotype in *Spink5* ^*-/-*^ mice. Here, we identified Klk5 as the major determinant of NS pathology. Solely by deleting *Klk5* gene, we successfully demonstrated reversal of both desquamating and inflammatory manifestations of NS. These were accompanied by drastic improvement of skin barrier defect, restoration of normal epidermal differentiation and epidermal ultrastructure. Our data identified KLK5 as a new target for drug development in NS, thus setting the foundation for designing the first targeted therapy against NS. NS shares several biological features and proteolytic unbalance with other inflammatory skin diseases such as atopic dermatitis, rosacea, and psoriasis. An increasing population suffers from these frequent skin diseases. Our findings could therefore have implication in the treatment of these common and disabling diseases.

## Introduction

The epidermis is a stratified epithelium that prevents from dehydration, excludes toxins and microbes, protects from mechanical injury, and participates in immune responses. The main barrier is provided by the *stratum corneum*, the outermost epidermal layer composed of multiple layers of terminally differentiated keratinocytes (mummified corneocytes) embedded in a lipid matrix [1,2]. Netherton Syndrome (NS, OMIM 256500) is a rare and severe autosomal recessive skin disease characterized by extensive skin desquamation, inflammation, multiple allergies, atopic manifestations and hair shaft defects [3,4]. There is currently no satisfactory treatment for NS which is a complex systemic disease with multiple effects, but only palliative treatments for management of skin infections, reduction of itching and pain [5,6]. Previously, we established that NS is caused by loss-of-function mutations in *SPINK5* [7], encoding LEKTI (lymphoepithelial Kazal-type inhibitor), a multidomain protease inhibitor. LEKTI has been shown to inhibit several members of the KLK serine protease family (KLK5, KLK7 and KLK14; [8,9]). The absence of LEKTI in NS results in unopposed KLKs activities and aberrantly increased epidermal proteolysis [8,10,11,12]. *Spink5* ^*-/-*^ mice recapitulate a phenotype highly reminiscent of NS, replicating cutaneous and inflammatory aspects of the disease [12,13,14,15,16]. Similarly to what has been observed in NS patients, *Spink5* ^*-/-*^ epidermis displays unopposed Klk5 and Klk7 protease activities [12]. *In vitro* studies showed that KLK5, KLK7 and KLK14 contribute to desquamation by degrading desmosomal cadherins such as Desmoglein 1 (Dsg1) and Desmocollin-1 (Dsc1) [9,17]. According to the current state-of-the art hypothesis, pro-KLKs are synthesized and activated in the *stratum granulosum* and active KLK enzymes are rapidly complexed with LEKTI, thus preventing premature degradation of desmosomes at the *stratum corneum*/*stratum granulosum* interface [8,11,18]. KLK-LEKTI complexes diffuse to the outer *stratum corneum* where the acidic microenvironment causes the release of active KLKs which cleave corneodesmosomal proteins in the most superficial layers of the *stratum corneum*. This ensures the finely-tuned regulation of the desquamation process. Two scenarios aim to explain the modes of proKLK activation in the skin. It was assumed that proKLK5 was autoactivated and that mature KLK5 activates other KLK zymogens. However, recent evidence indicates that proKLK5 can be activated by the transmembrane protease matriptase or by mesotrypsin which can both activate proKLK7 [8,19,20]. Thus, KLK5 is a hypothesized key regulator of physiological proteolysis in the epidermis and substantial evidence implicates its aberrant hyperactivity in overdesquamating and inflammatory skin diseases, like NS and atopic dermatitis [21]. In NS, loss of LEKTI causes premature degradation of desmosomes leading to abnormal detachment of the *stratum corneum* from the granular layer [12,14]. Epidermal hyperplasia, abnormal distribution of differentiation markers, increased Filaggrin (Flg) processing and lipid defects are also observed in NS skin [12,22]. In addition, unopposed proteolytic activity propagates sustained activation of pro-inflammatory and pro-signaling pathways, including the KLK5-PAR2-TSLP (thymic stromal lymphopoietin) axis [15]. Knocking-out Par-2 in *Spink5* ^*-/-*^ mice results in a dramatic decrease in TSLP expression at embryonic day 19.5 but these mice still display *stratum corneum* detachment. *Spink5* ^*-/-*^ */Par2* ^*-/-*^ grafted skin shows an inflammatory phenotype probably resulting from *stratum corneum* detachment [16]. To assess the role of KLK5 in NS, we recently developed a transgenic murine model overexpressing human KLK5 in the granular layer of the epidermis [23]. These animals reproduced major features of NS, including increased proteolytic activity in the skin, a severe skin barrier defect with cutaneous and systemic allergy and inflammation, identifying KLK5 as an important contributor of NS pathogenesis.

In this study, we developed a new murine model in which *Klk5* and *Spink5* were both inactivated to assess whether Klk5 knockout is sufficient to reverse the NS phenotype in *Spink5* ^*-/-*^ mice. This study allows a broader characterization of NS skin inflammation and reveals that KLK5 inactivation is sufficient to correct the cutaneous phenotype manifested in *Spink5* ^*-/-*^ newborn mice. These findings illustrate the crucial role of protease regulation in skin homeostasis and establish KLK5 inhibition as a major target for drug development for NS.

## Results

### Knockout of Klk5 expression reverses skin and whiskers anomalies

**Fig 1:**
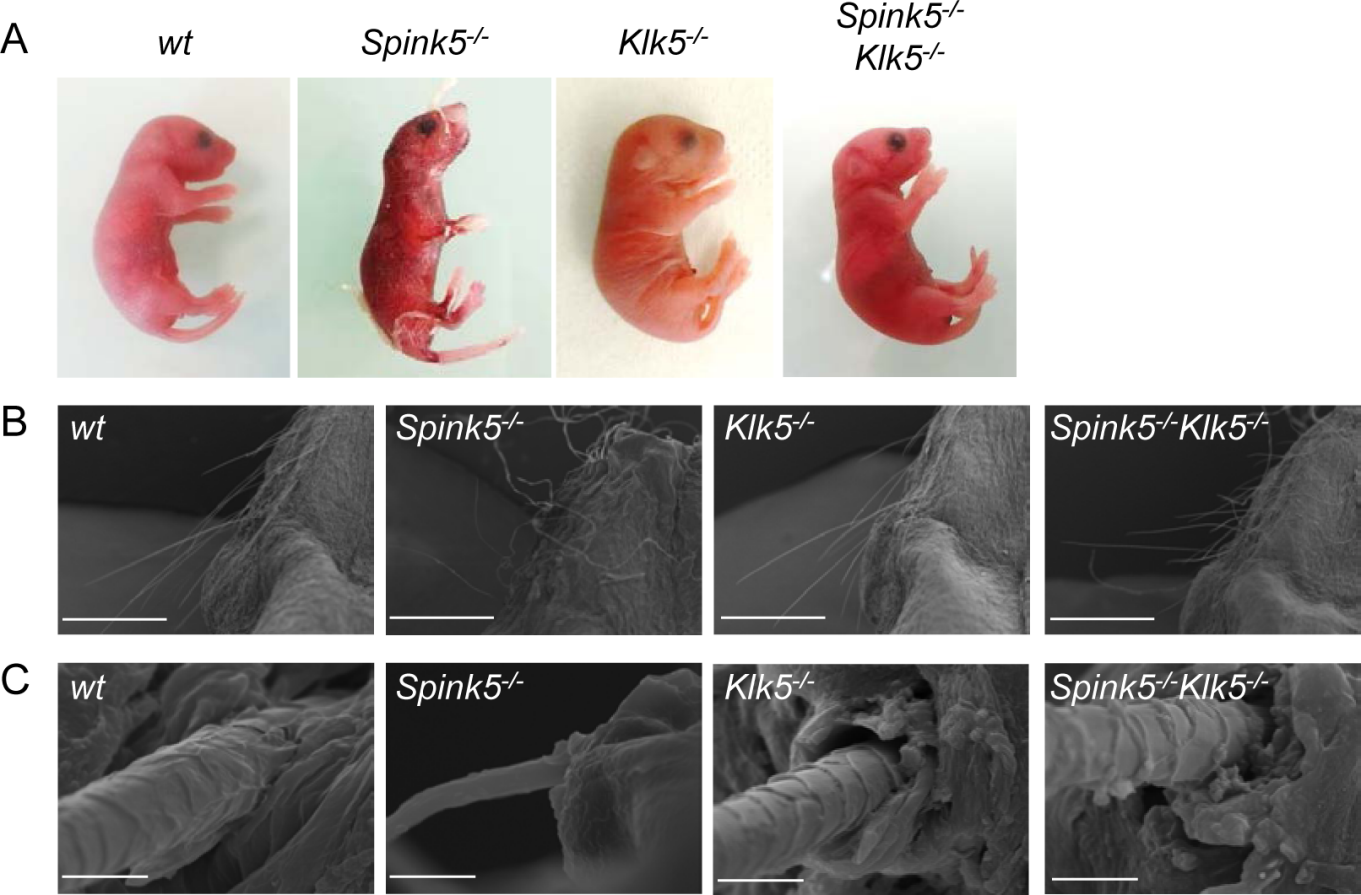
Loss of *Klk5* expression reverses skin and whisker anomalies in *Spink5* ^*-/-*^ mice. (**A**) Macroscopic appearance of wt, *Spink5* ^*-/-*^, *Klk5* ^*-/-*^, *Spink5* ^*-/-*^ *Klk5* ^*-/-*^ mice. Photos were taken 30 h after birth for wt, *Klk5* ^*-/-*^ and *Spink5* ^*-/-*^ *Klk5* ^*-/-*^ mice and 5 h for *Spink5* ^*-/-*^ mice; (**B**) Microscopic appearance of muzzle area (scale bar 1mm) and (**C**) whisker ultrastructure by scanning electron microscopy (SEM). Scale bar, 10μm.

We and others have previously reported that *Spink5* ^*-/-*^ mice show neonatal lethality due to a major skin barrier defect [12,13,14]. To investigate the contribution of Klk5 in the *Spink5* ^*-/-*^ phenotype, we have generated *Klk5* ^*-/-*^ mice on a C57BL/6 background (S1 Fig). Mice are viable, fertile and do not show any macroscopic cutaneous phenotype. *Klk5* ^*-/-*^ mice were intercrossed with *Spink5* ^*+/-*^ on the same pure genetic background and *Klk5* ^*+/-*^ *Spink5* ^*+/-*^ double heterozygotes were intercrossed to generate *Spink5* ^*-/-*^ *Klk5* ^*-/-*^ double knockout mice that were identified using a PCR-based genotyping strategy (S1 Table). We confirmed the absence of detectable levels of *Klk5* and *Spink5* mRNA by quantitative RT-PCR in *Spink5* ^*-/-*^ *Klk5* ^*-/-*^ skin (S2 Fig). *Spink5* ^*-/-*^ mice developed desquamating lesions within 1 hour from birth and died shortly after (<5 h) (Fig 1A). They also displayed vibrissae defects ranging from complete absence to rare and disorganized whiskers [12,13,14]. In striking contrast, newborn *Spink5* ^*-/-*^ *Klk5* ^*-/-*^ mice displayed no apparent cutaneous phenotype, neither signs of skin desquamation nor inflammation and were overall indistinguishable from wild-type (wt) mice, except that they grew shorter whiskers (Fig 1A and 1B). As shown in Fig 1C, the microstructure of *Spink5* ^*-/-*^ *Klk5* ^*-/-*^ whiskers was nearly identical to those of the wt animals and very different from the short, thin, and disorganized whiskers observed in *Spink5* ^*-/-*^ mice. Neonates from all genotypes were fed normally as milk could be visualized in their stomach and had normal weight (≈1.30 g for all genotypes) at birth.

### Ablation of Klk5 expression remarkably improves epidermal function

**Fig 2:**
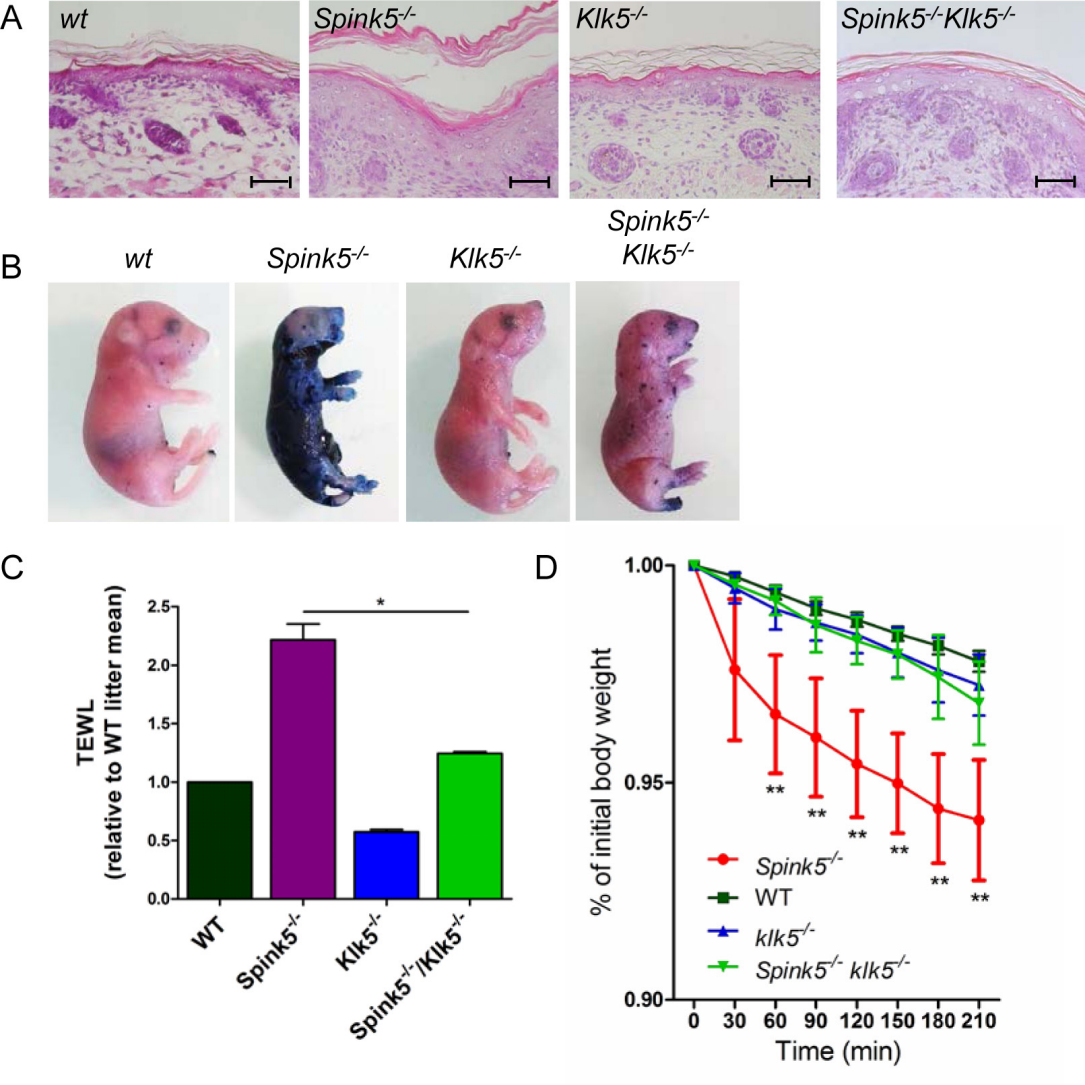
Skin barrier function is restored by Klk5 knockout. (**A**) Hematoxylin/eosin/safranin (HES) stained skin sections show no *stratum corneum* detachment and normal thickness of the living layer in the epidermis of *Spink5* ^*-/-*^ *Klk5* ^*-/-*^ (taken post-natally at 30 h) as opposed to *Spink5* ^*-/-*^ (at 5 h) characterized by extensive *stratum corneum*/*stratum granulosum* separation and acanthosis; Scale bar: 50μm (**B**) Skin barrier defect is remarkably improved in *Spink5* ^*-/-*^ *Klk5* ^*-/-*^ as demonstrated by the toluidine blue dye penetration assay. The dye is excluded from entering the body of the *Spink5* ^*-/-*^ *Klk5* ^*-/-*^ mouse in sharp contrast to the deep blue stain of *Spink5* ^*-/-*^; (**C**) Transepidermal water loss (TEWL) assay. TEWL values are significantly lower in *Spink5* ^*-/-*^ *Klk5* ^*-/-*^ as compared to *Spink5* ^*-/-*^ and similar to wt animals, reflecting normalization of the skin barrier defect on the Lekti-deficient background solely by Klk5 deletion. *p<0.05 (Mann-Whitney U test); (**D**) TEWL values over time in wt (n = 9), *Spink5* ^*-/-*^ (n = 9), *Klk5* ^*-/-*^ (n = 10) and combined *Spink5* ^-/-^ *Klk5* ^-/-^ deficient (n = 8) newborn mice at 37°C. Data are shown as mean ± s.e.m. (standard error of the mean). Stars on the graph represent statistics between *Spink5* ^*-/-*^ and *Spink5* ^-/-^ *Klk5* ^-/-^ at the different time points **p<0.01 (Mann-Whitney U test). Note that values for *Spink5* ^-/-^ *Klk5* ^-/-^ and *Klk5* ^*-/-*^ are not significantly different from wt whereas *Spink5* ^*-/-*^ values are significantly different from wt for all time points.

Defective skin barrier as observed in NS patients and in *Spink5* ^*-/-*^ mice results in the development of compensatory mechanisms in the epidermis leading to hyperkeratosis (thickening of the cornified layer) and acanthosis (thickening of the living layers) (Fig 2A) [12]. *Stratum corneum* detachment is also a characteristic feature of NS (Fig 2A). In contrast, histology examination of skin sections from *Spink5* ^*-/-*^ *Klk5* ^*-/-*^ mice showed neither acanthosis nor hyperkeratosis, nor microscopic separation of the *stratum corneum*/*stratum granulosum* (Fig 2A).

To investigate epidermal barrier function, we first examined the ability of the skin to prevent penetration of an external dye solution in a whole-mount assay. Toluidine blue dye permeability assay showed a major skin permeability defect in *Spink5* ^*-/-*^ as compared to wt and *Klk5* ^*-/-*^ animals. In contrast, only a few patches of dye penetration were seen in *Spink5* ^*-/-*^ *Klk5* ^*-/-*^ mice, indicating that deletion of *Klk5* in Lekti-deficient mice drastically improved epidermal barrier function (Fig 2B). The presence of these patches could be due to additional proteolytic activities which remain active and take over upon *Klk5* invalidation. Consistent with this result, *Spink5* ^*-/-*^ *Klk5* ^*-/-*^ mice exhibited significantly lower transepidermal water loss (TEWL) compared to *Spink5* ^*-/-*^ mice (Fig 2C). The remarkable improvement of skin functional integrity in *Spink5* ^*-/-*^ *Klk5* ^*-/-*^ animals was further confirmed by the absence of weight loss over time (210 minutes) at 37°C (Fig 2D).

### Klk5 knockout down-regulates aberrant protease activity

**Fig 3:**
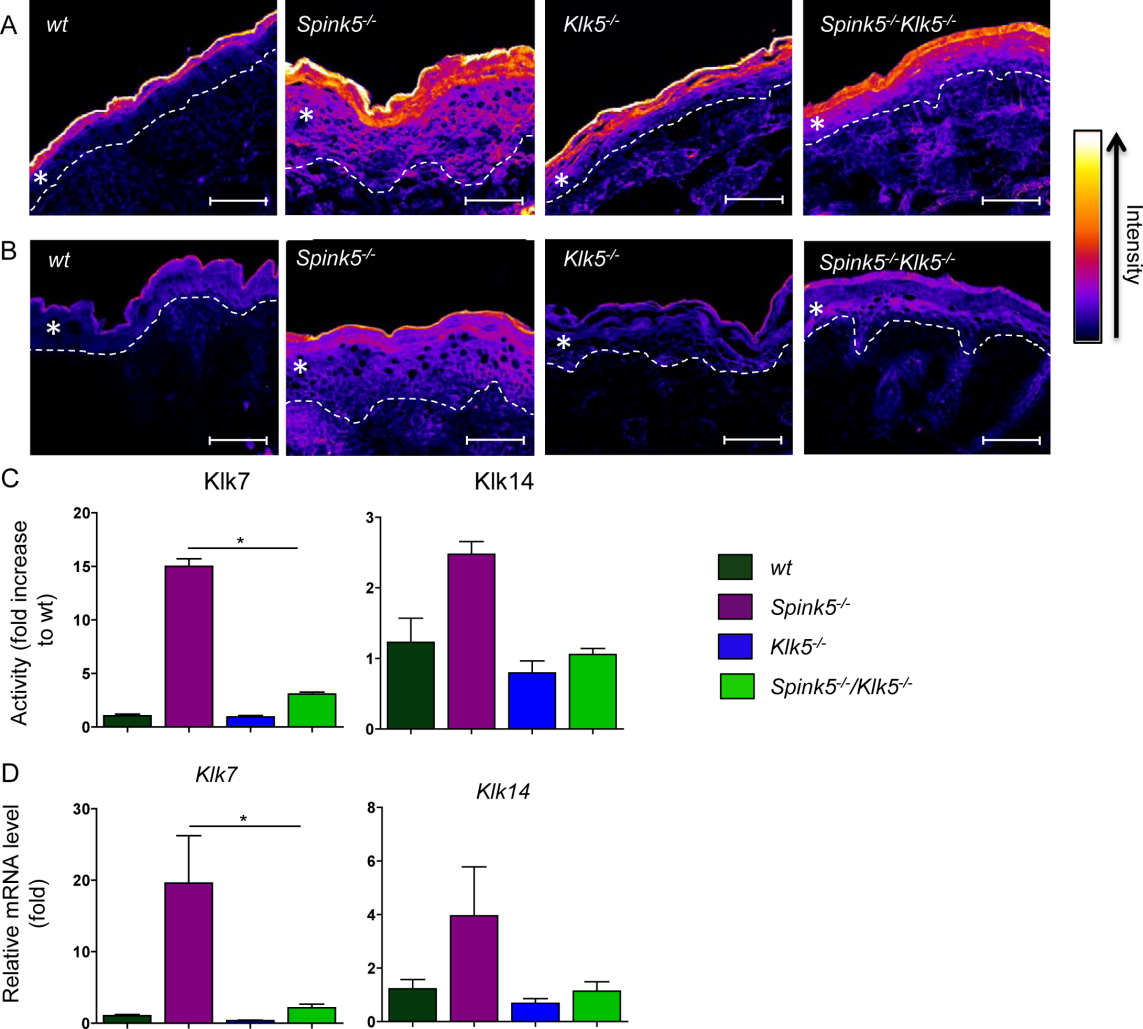
Klk5 is a key regulator of epidermal proteolysis. *In situ* zymography using fluorescence-quenched casein (**A)** or elastin (**B**). Skin tissue sections were prepared from wt, *Spink5* ^*-/-*^, *Klk5* ^*-/-*^, and *Spink5* ^*-/-*^ *Klk5* ^*-/-*^ mice. Ablation of *Klk5* expression highly reduced both caseinolytic and elastinolytic activities in the *stratum corneum* of *Spink5* ^*-/-*^ *Klk5* ^*-/-*^ as compared to *Spink5* ^*-/-*^, respectively. Fluorescence intensity data was transformed into a color gradient (as shown) using ImageJ software. Dashed white lines represent the dermal-epidermal junction; The asterisk (*) indicates the epidermis, scale bar: 25μm (**C**) Changes in proteolytic activity using colorimetric substrates that target different proteases. Activity in wt, *Spink5* ^*-/-*^ and *Spink5* ^*-/-*^ *Klk5* ^*-/-*^ was detected by measuring absorbance at 405 nm after overnight incubation with substrates for either KLK7 or KLK14. Data are shown as the mean ± SEM of duplicates for four mice per genotype; (**D**) Analysis of mRNA expression in skin by RT-qPCR of Klk7 and Klk14. Results show high expression of both Klks in *Spink5* ^*-/-*^. In *Spink5* ^*-/-*^ *Klk5* ^*-/-*^ skin, expression of Klk14 is equal to wt while Klk7 remained slightly higher. Data are shown as the mean ± s.e.m. of triplicate amplification for at least three mice per genotype. Results are normalized to wt mean (set as 1.0).

A characteristic feature of the *Spink5* ^*-/-*^ phenotype is aberrantly increased proteolysis in the skin, especially in the upper layers of the epidermis, leading to the separation of the *stratum corneum* from the *stratum granulosum* as a result of Dsg1 cleavage [12,13,14,20]. The overall proteolytic activity was visualized in skin sections by *in situ* zymography using quenched fluorescent casein and elastin substrates (Fig 3A and 3B, respectively). Very high proteolytic activities were detected in *Spink5* ^*-/-*^ skin sections in which caseinolytic activity predominated in the upper layers of the epidermis and diffused throughout the hyperplastic epidermis [20]. The overall caseinolytic activity in the epidermis of newborn *Klk5* ^*-/-*^ mice was not significantly reduced compared to the wt at birth (Fig 3A). In contrast, genetic knock out of *Klk5* had a very strong suppressing effect on caseinolytic activity in *Spink5* ^*-/-*^ *Klk5* ^*-/-*^ skin, as shown in Fig 3A, indicating a dominant role of Klk5 in NS pathology. To further explore proteolytic activity in *Spink5* ^*-/-*^ and *Spink5* ^*-/-*^ *Klk5* ^*-/-*^ skin, we used peptide substrates known to be cleaved by downstream Klk5 proteases (Klk7 and Klk14)[24,25]. Cleavage of Klk7 substrate was elevated by more than 14-fold in *Spink5* ^*-/-*^ skin compared to wt and drastically reduced in *Spink5* ^*-/-*^ *Klk5* ^*-/-*^, although it was still slightly elevated compared to wt (2-fold) (Fig 3C). Cleavage of Klk14 substrate was elevated by more than 2-fold in *Spink5* ^*-/-*^ skin compared to wt but was normalised in *Spink5* ^*-/-*^ *Klk5* ^*-/-*^ (Fig 3C). In addition Klk7 and Klk14 mRNA expression was increased in *Spink5* ^*-/-*^ skin and down-regulated in *Spink5* ^*-/-*^ *Klk5* ^*-/-*^ (Fig 3D). The elastinolytic activity was also significantly increased in the epidermis of *Spink5* ^*-/-*^ and markedly reduced in *Klk5* ^*-/-*^ and *Spink5* ^*-/-*^ *Klk5* ^*-/-*^ epidermis (Fig 3B).

### Klk5 inactivation restores desmosome integrity, epidermal architecture and differentiation

**Fig 4:**
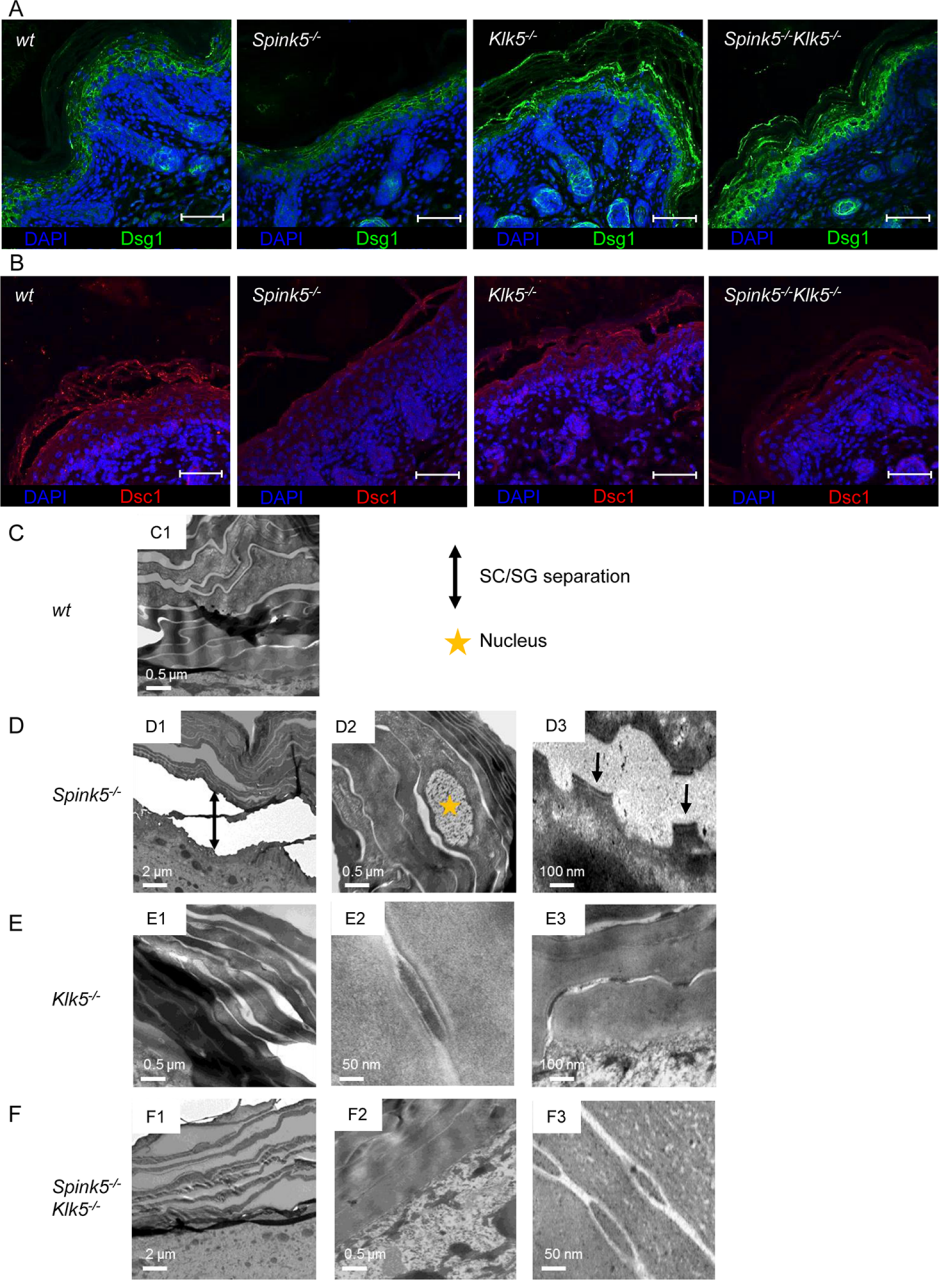
The ultrastructural architecture of *Spink5* ^*-/-*^ epidermis is normalized by *Klk5* knockout. (**A**) Immunostaining on skin section shows restored Dsg1 in *Spink5* ^*-/-*^ *Klk5* ^*-/-*^ compared to *Spink5* ^*-/-*^ consistent with diminished pathological proteolysis observed in Fig 3, scale bar: 25μm (**B**) Immunostaining on skin section shows partial restoration of Dsc1 in *Spink5* ^*-/-*^ *Klk5* ^*-/-*^ compared to *Spink5* ^*-/-*^, scale bar: 25μm. (**C**) Transmission electron micrographs of skin ultrasections from: wt (**C1-2**), *Spink5* ^*-/-*^ (**D1-3**), *Klk5* ^*-/-*^ (**E1-3**), and *Spink5* ^*-/-*^ *Klk5* ^*-/-*^ (**F1-3**) mice. Nuclei (depicted with star in **D2**) present in the *stratum corneum* of *Spink5* ^*-/-*^ denote incomplete differentiation (parakeratosis). Separation of desmosomes in *Spink5* ^*-/-*^ is evident, as indicated by arrows (**D3**). Of note, also the well-organized compact desmosomes in *Klk5* ^*-/-*^ and *Spink5* ^*-/-*^ *Klk5* ^*-/-*^ skin sections (**E2-3, F3**).

Several studies have shown that KLK5 is able to degrade Dsg1 and Dsc1 *in vitro*, thus contributing to the detachment of superficial corneocytes during desquamation [17,26,27]. Dsg1 is a major desmosomal cadherin which is cleaved in the most superficial layers of the epidermis during the desquamation process. Previous studies established that Dsg1 and Dsc1 are degraded *in vivo* by enhanced proteolytic activity in NS patients, in *Spink5* ^*-/-*^ and Tg-*KLK5* mice [12,23,28]. As shown in Fig 4A, Dsg1 is drastically reduced in *Spink5* ^*-/-*^, is increased in *Klk5* ^*-/-*^ epidermis and is restored in *Spink5* ^*-/-*^ *Klk5* ^*-/-*^ mice compared to wt mice (Fig 4A and S5A Fig). Dsc1 expression is also decreased in *Spink5* ^*-/-*^, is comparable to wt mice in *Klk5* ^*-/-*^ epidermis and is partially restored in *Spink5* ^*-/-*^ *Klk5* ^*-/-*^ mice (Fig 4B and S5B Fig). These results are consistent with reduced overall proteolytic activity and absence of *stratum corneum* detachment as seen in *Spink5* ^*-/-*^ *Klk5* ^*-/-*^ epidermis compared to *Spink5* ^*-/-*^.

Transmission electron microscopy (TEM) showed remnant nuclei in corneocytes (parakeratosis) in *Spink5* ^*-/-*^ whereas no nuclei were seen in the *stratum corneum* of wt, *Klk5* ^*-/-*^ and *Spink5* ^*-/-*^ *Klk5* ^*-/-*^ animals (Fig 4C1, 4D2, 4E1 and 4F1) [12,29,30]. TEM also showed *stratum corneum/stratum granulosum* separation with split-desmosomes in *Spink5* ^*-/-*^, which were not seen in wt, *Klk5* ^*-/-*^ and *Spink5* ^*-/-*^ *Klk5* ^*-/-*^ (Fig 4C1, 4D1, 4D3, 4E1, 4F1 and 4F2). In contrast, a compact *stratum corneum* structure with intact desmosomes and corneodesmosomes was seen in *Spink5* ^*-/-*^ *Klk5* ^*-/-*^ as opposed to *Spink5* ^*-/-*^ animals (Fig 4C1, 4D3, 4E1 and 4E2). Notably, the structure of corneodesmosomes in both *Klk5* ^*-/-*^ and *Spink5* ^*-/-*^ *Klk5* ^*-/-*^ skin appeared more compact and dense than in wt skin (Fig 4E2, 4E3 and 4F3). In addition, *Klk5* ^*-/-*^ and *Spink5* ^*-/-*^ *Klk5* ^*-/-*^ skin showed an increased number of corneodesmosomes compared to wt (S3A Fig), with a significantly enhanced number of uncleaved corneodesmosomes (S3B Fig). These observations are consistent with decreased epidermal proteolysis and higher Dsg1 expression in *Klk5* ^*-/-*^ and *Spink5* ^*-/-*^ *Klk5* ^*-/-*^ and also identify Dsg1 as an *in vivo* substrate of Klk5 (Fig 4A).

**Fig 5:**
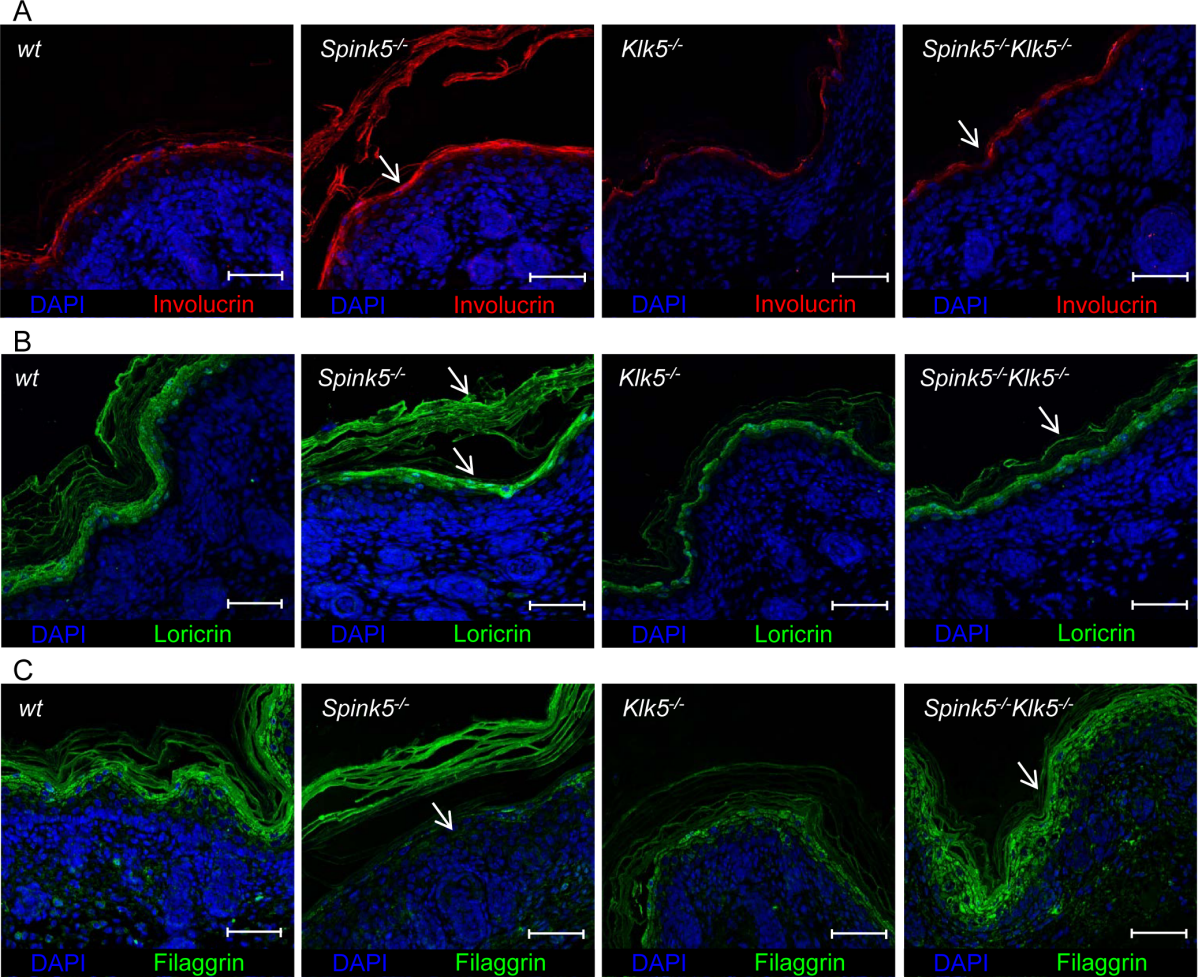
*Spink5* ^*-/-*^ *Klk5* ^*-/-*^ skin does not show evidence of abnormal epidermal differentiation. (**A, B**) IHF analyses show normalized Involucrin and Loricrin expression in *Spink5* ^*-/-*^ *Klk5* ^*-/-*^ skin compared to *Spink5* ^*-/-*^ as indicated by arrows; scale bar: 25μm (**C**) Deletion of Klk5 blocks Flg degradation. Immunostaining of Flg extends to the granular layer and the *stratum corneum* in wt, *Klk5* ^*-/-*^, *Spink5* ^*-/-*^ *Klk5* ^*-/-*^ while it is drastically reduced in the granular layer and restricted to the detached *stratum corneum* in *Spink5* ^*-/-*^ mice as indicated by arrows; scale bar: 25μm.

Skin from NS patients and murine models displays several features of impaired epidermal differentiation and cornification [12,22]. Involucrin and loricrin expression, which is enhanced in *Spink5* ^*-/-*^ mice, show a pattern of expression similar to wt in *Spink5* ^*-/-*^ *Klk5* ^*-/-*^ mice (Fig 5A and 5B and S6A and S6B Fig).

*Spink5* ^*-/-*^ skin exhibits accelerated degradation of Flg and lipid defects [12,22]. In wt and *Klk5* ^*-/-*^ mice, Flg immunostaining is detected in the granular layers and *stratum corneum* (Fig 5C and S6A Fig). In *Spink5* ^*-/-*^ skin, Flg immunostaining is drastically reduced, almost absent in the granular layer and restricted to detached *stratum corneum*. The pattern of Flg expression is fully restored in *Spink5* ^*-/-*^ *Klk5* ^*-/-*^ skin (Fig 5C and S6A Fig). Consistent with these results, Flg western blot analysis showed that, in contrast to *Spink5* ^*-/-*^ skin which displayed reduced or no detectable high molecular weight forms of Flg, *Spink5* ^*-/-*^ *Klk5* ^*-/-*^ skin did not reveal evidence for increased proteolytic cleavage of pro-Flg (S4A Fig). The amounts of cholesterol, neutral and polar lipids were visualized by filipin and Nile red staining, respectively (S4B and S4C Fig). Both stains revealed linear lipid structures corresponding to intercellular spaces in wt animals. In *Spink5* ^*-/-*^, a pearl-like lipid distribution was observed which was more pronounced with filipin. This staining pattern was also reported in skin sections from NS patients [22]. Only few cholesterol deposits were seen in the *stratum corneum* of wt and *Klk5* ^*-/-*^ mice. Both stains revealed that this abnormal pearl-like pattern was alleviated in *Spink5* ^*-/-*^ *Klk5* ^*-/-*^ epidermis, suggesting that elimination of Klk5 partially restored corneocyte lipid envelope formation. In conclusion, restoration in large part of epidermal differentiation and architecture with lack of *stratum corneum* detachment in *Spink5* ^*-/-*^ *Klk5* ^*-/-*^ allowed recovery of epidermal barrier function.

### Klk5 loss prevents cutaneous inflammation

**Fig 6:**
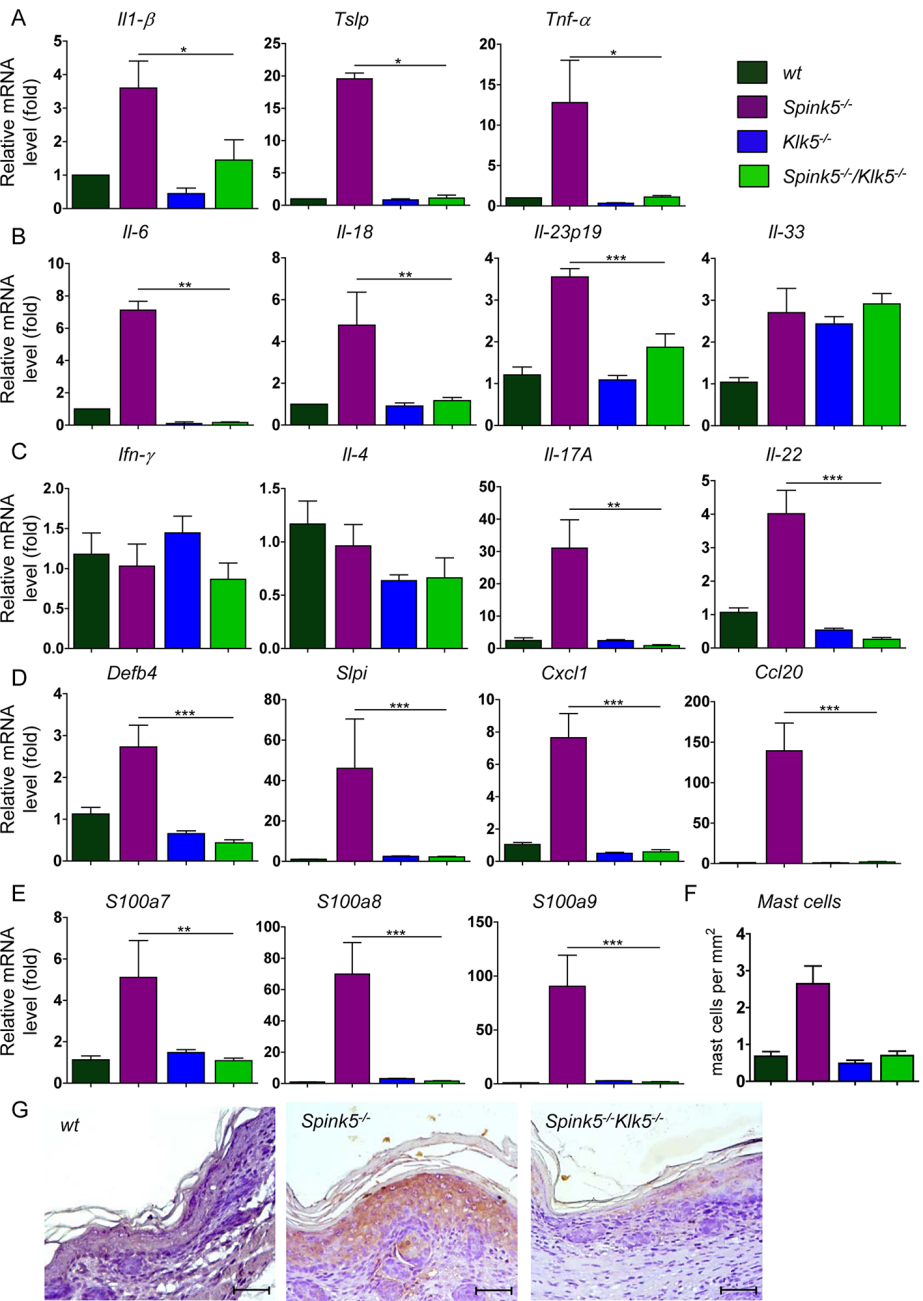
*Klk5* deletion results in remarkable suppression of the inflammatory phenotype of NS. (**A-E**). Results show remarkable abrogation of pro-allergic and pro-inflammatory cytokines quantified by RT-qPCR. (**A**) Il-1β, Tslp, Tnf-α; (**B**) Il-6, Il-18, the type 17 promoting cytokine Il-23p19; (**C**) Ifn-γ, Il-4, Il-17A, Il-22; (**D**) Th17 regulated genes Defb4, Slpi, Cxcl1 and Ccl20; (**E**) Th17/22 regulated genes s100a7, s100a8, s100a9. Data are shown as the mean ± s.e.m. of triplicate amplification for at least five mice per genotype. Results are normalized to wt mean (set as 1.0). *p<0.05, **p<0.005, ***p<0.001 (Mann-Whitney U test). (**F**) Quantification of mast cells based on toluidine blue staining of skin tissue (mast cells stain purple). An increased number of mast cells was detected in *Spink5* ^*-/-*^ dermis; (**G**) IHC analysis shows abolishment of Tslp expression in *Spink5* ^*-/-*^ *Klk5* ^*-/-*^ skin as compared to *Spink5* ^*-/-*^; Scale bar: 50μm.

*Spink5* ^*-/-*^ mice and NS patients show severe inflammation of the skin [12,13,14]. Pro-inflammatory signaling in Lekti-deficient epidermis involves activation of the PAR2-NF-κB axis by unopposed KLK5 activity leading to overexpression of TSLP (thymic stromal lymphopoietin), enhanced TNF-α, intracellular adhesion molecule (ICAM-1) and IL-8 expression. Increased proteolytic processing of the cathelicidin precursor also contributes to skin inflammation through the release of antimicrobial and pro-inflammatory peptides [15,31,32]. Consistent with these observations, *Spink5* ^*-/-*^ skin grafts on nude mice show increased expression of pro-inflammatory and pro-allergic cytokines and exhibit an inflammatory infiltrate composed of mast cells and eosinophils [15]. In contrast to *Spink5* ^*-/-*^, *Spink5* ^*-/-*^ *Klk5* ^-/-^ mice showed no redness of their skin, indicating that skin inflammation associated with Lekti-deficiency was remarkably inhibited by the sole elimination of Klk5 activity (Fig 1A). Consistently, targeted transcript analysis using qRT-PCR showed that several pro-allergic and pro-inflammatory cytokine-encoding mRNAs previously reported to be enhanced in *Spink5* ^*-/-*^[15], were markedly reduced in *Spink5* ^*-/-*^ *Klk5* ^*-/-*^ skin (Fig 6)[15]. Specifically, expression of Tslp, a major pro-Th2 cytokine, was strongly diminished in *Spink5* ^*-/-*^ *Klk5* ^*-/-*^ skin, at the mRNA level and at the protein level as verified by immunohistochemistry depicted in Fig 6A and 6G and S6D Fig. Tnf-α, Il-1β, Il-6, and Il-18 pro-inflammatory cytokine expression was elevated in *Spink5* ^*-/-*^ and significantly down- regulated in *Spink5* ^*-/-*^ *Klk5* ^*-/-*^ skin (Fig 6A and 6B). Down-regulation of these inflammatory markers correlated with normalized Par-2 expression in the epidermis in *Spink5* ^*-/-*^ *Klk5* ^*-/-*^ (S5A and S6C Figs). Among the Il-25 and Il-33 alarmins involved in Th2 responses, only Il-33 showed a moderate increase in both *Spink5* ^*-/-*^ and *Spink5* ^*-/-*^ *Klk5* ^*-/-*^ (Fig 6B and S5B Fig). We next investigated the expression of molecules known to attract immune cells to the skin. Ccl17/Tarc and CCL22/Mdc are major pro-Th2 mediators that can recruit pro-allergic Th2 cells *via* the chemokine receptor CCR4 to the skin [33]. In *Spink5* ^*-/-*^ skin, Ccl17 and Ccl22 were elevated while Ccl22 was down regulated in *Spink5* ^*-/-*^ *Klk5* ^*-/-*^ (S5C Fig). The expression of Ccl8, known to attract actors of allergy and inflammation, was elevated in both *Spink5* ^*-/-*^ and *Spink5* ^*-/-*^ *Klk5* ^*-/-*^ skin (S5C Fig), whereas Ccl20, a chemoattractant for CCR6^+^ cells including dendritic cells and Th17 cells, showed a significant increase only in *Spink5* ^*-/-*^ skin (Fig 6D). We further characterized the nature of the inflammatory responses developed in *Spink5* ^*-/-*^ and compared it to *Spink5* ^*-/-*^ *Klk5* ^*-/-*^ skin (Fig 6B). We found no evidence for Th1 (Ifn-γ) or Th2 (Il-4 and Il-13) responses in the skin of all genotypes (Fig 6C and S5D Fig). Specifically, of the genes up- regulated by Th1/IFNγ, including *Ccl5*, *Cxcl9*, *Cxcl10* and *Cxcl11*, only Cxcl9 and Cxcl10 showed slightly increased expression in *Spink5* ^*-/-*^ skin (S5E Fig). In contrast, expression of type 17 promoting cytokine Il-23 p19 was enhanced in *Spink5* ^*-/-*^ newborn skin. Both Th17-type cytokines Il-17-A and Il-22 were significantly increased in *Spink5* ^*-/-*^ and were not detectable in *Spink5* ^*-/-*^ *Klk5* ^*-/-*^ skin (Fig 6C). Additionally, genes known to be up- regulated by Il-17 such as *Defb4*, *Slpi* and *Cxcl1* showed elevated expression at the mRNA level in *Spink5* ^*-/-*^ skin only (Fig 6D) [34]. IL-17 and IL-22 are also known to up- regulate S100A7, S100A8 and S100A9 expression in human keratinocytes [34,35]. Increased levels of Il-17 and Il-22 transcripts in *Spink5* ^*-/-*^ skin coincided with a significant increase in S100a7, S100a8 and S100a9 mRNA expression (Fig 6E). Finally, mast cells and neutrophils were infiltrating *Spink5* ^*-/-*^ skin, which is consistent with the recruitment of these cells by this inflammatory environment (Fig 6F, S5F and S6E Figs).

Collectively, these data demonstrate that Klk5 ablation is sufficient to block the development of cutaneous inflammation and allergy in the context of Lekti-deficiency. Klk5 ablation prevents the expression of pro-inflammatory and pro-allergic cytokines, the infiltration of immune cells and the development of the Th17 inflammatory axis.

## Discussion

Our results establish that Klk5 deletion in *Spink5* ^*-/-*^ mice leads to remarkable reduction of aberrant epidermal proteolysis and inflammation, restores normal differentiation and drastically improves skin barrier structure and function. They provide *in vivo* evidence that KLK5 is a key actor and a major therapeutic target in NS.

NS is a complex disease with severe skin inflammation, scaling and constant allergic manifestations for which clinicians and scientists do not have a complete understanding. We and others previously characterized *Spink5*-deficient mice, which display key features of NS but die shortly after birth [12,13,14]. Although *Spink5* ^*-/-*^ *Klk5* ^*-/-*^ mice survive the neonatal phase and do not die within few hours as *Spink5* ^*-/-*^ do, they do not survive as long as wt animals. The number of patches of dye penetration observed in *Spink5* ^*-/-*^ *Klk5* ^*-/-*^ did not increase with time, indicating that the mice did not die from a skin barrier defect (S7 Fig). This observation argues for unidentified defaults in *Spink5* ^*-/-*^ that are not completely or partially corrected by *Klk5* knockout and which could be identified because *Spink5* ^*-/-*^ *Klk5* ^*-/-*^ mice did not die from dehydration within the first hours of life as *Spink5* ^*-/-*^ did. The cause of death of those animals is currently under investigation. Up to now, studies in NS murine models and in patients have unraveled some of the mechanisms involved in the pathophysiology. In NS skin, loss of LEKTI expression leads to unopposed activity of several proteases [12,22,28]. While increased caseinolytic activity was detected in *Spink5* ^*-/-*^ skin, we show that knockout of *Klk5* had a drastic repressing effect on caseinolytic activity in *Spink5* ^*-/-*^ *Klk5* ^*-/-*^ skin, confirming the hypothesized role of Klk5 as an initiator of the epidermal proteolytic cascade. The fact that proteolytic activity in *Spink5* ^*-/-*^ *Klk5* ^*-/-*^ is slightly more elevated than in wt skin reveals that other proteases, independently of Klk5 are still active. Using peptide substrates, we identified Klk7 activity as being still slightly elevated compared to wt, possibly as a result of activation by matriptase or mesotrypsin [8,19,20]. Interestingly, Klk7 and Klk14 mRNA levels were increased in *Spink5* ^*-/-*^ skin and were normalized in *Spink5* ^*-/-*^ *Klk5* ^*-/-*^. Therefore it appears that *Klk5* knockout in Lekti-deficient animals prevents increased Klk expression driven by skin inflammation in *Spink5* ^*-/-*^ mice and aberrant proteolytic activity of Klk5 target proteases (Klk7, Klk14 and Ela2) [36,37]. This new murine model in which Klk5 is lacking will be instrumental to decipher the network of activated proteases in NS regardless Klk5. Of note, suppression of caseinolytic activity by *Klk5* knockout on Lekti-deficient background was significantly more pronounced than the one previously reported in double knock-out mice for *Spink5* and matriptase which is nevertheless involved in Klk5 activation [20]. Elastinolytic activity was also markedly reduced in *Spink5* ^*-/-*^ *Klk5* ^*-/-*^ epidermis compared to *Spink5* ^*-/-*^, supporting the notion that Klk5 contributes to pro-ELA2 activation, as suggested by *in vitro* [22] and *in vivo* studies in transgenic KLK5 mice [23].

NS patients suffer from a profound skin barrier defect. Macroscopic examination of *Spink5* ^*-/-*^ newborn mice showed a major peeling of the skin whereas *Spink5* ^*-/-*^ *Klk5* ^*-/-*^ mice were indistinguishable from wt littermates. Loss of *Klk5* leads to skin barrier function recovery as illustrated by the absence of water loss and dye penetration in *Spink5* ^*-/-*^ *Klk5* ^*-/-*^ mice. Histological examination of *Spink5* ^*-/-*^ skin revealed epidermal hyperplasia, hyperkeratosis and parakeratosis, typical features of NS skin, consistent with compensatory hyperproliferative mechanisms secondary to skin barrier defects [30]. Another NS skin hallmark is abnormal detachment of the *stratum corneum* from the underlying granular layer through desmosomal cleavage [12,28]. All these skin abnormalities were not seen after *Klk5* deletion in Lekti deficient mice. Several studies have shown that KLK5 is able to degrade Dsg1 and Dsc1 *in vitro* [9,17]. In *Spink5* ^*-/-*^ mouse skin and in NS patients, increased activity of KLK5 and its target proteases leads to desmosomal cleavage through Dsg1 and Dsc1 degradation [12,23,28]. Here we show that Klk5 deletion totally prevented abnormal desmosomal cleavage in *Spink5* ^*-/-*^ skin. Additionally, increased Dsg1 and increased number of compact and uncleaved corneodesmosomes in *Klk5* ^*-/-*^ confirms the crucial role of Klk5 in the desquamation process *in vivo* and emphasizes the importance of protease regulation in skin homeostasis. Of note, Il-22 expression was increased in *Spink5* ^*-/-*^ skin and was normalized in *Spink5* ^*-/-*^ *Klk5* ^*-/-*^. IL-22 is known to induce epidermal hyperplasia and to impair keratinocyte differentiation, and is increased in chronic stages of atopic dermatitis and in psoriasis [34]. Il-22 could therefore contribute to acanthosis and abnormal differentiation in NS which do not develop in *Spink5* ^*-/-*^ *Klk5* ^*-/-*^. Consistent with this hypothesis, elevated Il-22 levels were found in mice overexpressing KLK5 in the epidermis [23]. The major alterations in epidermal terminal differentiation markers and Flg processing of *Spink5*^*-/-*^ skin were absent in *Spink5*^*-/-*^*Klk5*^*-/-*^. Abnormal expression of these proteins in *Spink5*^*-/-*^ skin could reflect proteolytic degradation, impaired protein processing or impaired differentiation. Involucrin and loricrin, two protein precursors of the epidermal cornified envelope, were overexpressed in *Spink5* ^*-/-*^ newborns [12] and showed normal expression in *Spink5* ^*-/-*^ *Klk5* ^*-/-*^. Profilaggrin processing into Filaggrin monomers was increased in *Spink5* ^*-/-*^ neonates [12,22], but displayed a normal pattern in *Spink5* ^*-/-*^ *Klk5* ^*-/-*^. Finally, Flg expression, which is drastically reduced in *Spink5* ^*-/-*^, was strongly expressed in the granular layer and *stratum corneum* in *Spink5* ^*-/-*^ *Klk5* ^*-/-*^. KLK5 and Ela2 contribute to pro-Flg degradation *in vivo* and *in vitro* [22,38]. The observation that Klk5 loss in *Spink5* ^*-/-*^ *Klk5* ^*-/-*^ leads to a strong decrease of elastinolytic activity confirms the important role of Klk5 and its target protease Ela2 in pro-Flg processing and to a larger extent in epidermal differentiation and skin barrier integrity.

Studies using *Spink5* ^*-/-*^ skin grafting experiments and embryo skin have shown increased expression of several pro-inflammatory and pro-allergic molecules such as Il-1β, Tnf-α, Icam-1, Tslp, Ccl17 (Tarc) and Ccl22 (Mdc) [15]. In NS patients, an intrinsic mechanism takes place in keratinocytes and leads to increased expression of TSLP, TNF-α, IL-8 and ICAM-1 as a result of PAR2 activation by active KLK5 [15]. Our study confirms the results previously obtained in another genetic background (C57Bl/6 in our study versus mixed C57Bl/6 and FVB), further characterizes *Spink5* ^*-/-*^ skin inflammation and investigates the effect of *Klk5* inactivation on NS inflammatory profile. TSLP expression, which is increased in NS skin and in lesional atopic skin, plays a major role in the induction of Th2 pro-allergic response [15,39]. In *Spink5* ^*-/-*^ newborn skin, although we measured elevated expression of Tslp and moderate increased expression of Ccl17 and Ccl22, we found no evidence of Th2 response (Il-4, Il-13). Tslp expression was entirely abolished in *Spink5* ^*-/-*^ *Klk5* ^*-/-*^ skin, confirming a role of Klk5 in Tslp induction. Our results clearly show that Klk5 knockout totally blocks the Par-2 mediated inflammation in *Spink5* ^*-/-*^ newborn, in addition to Klk5 direct action on desmosomal cleavage. NS patients suffer from multiple atopic features such as eczematous like lesions, allergic asthma, allergic rhinitis, urticaria and angioedema [40]. The development of Th2 environment in Lekti-deficient skin in NS patients and in grafted *Spink5* ^*-/-*^ mouse skin could be in part due to an “intrinsic” TSLP production by keratinocytes and infiltrating cells, but also to the skin barrier defect resulting from *stratum corneum* detachment and Flg degradation, allowing exogenous proteases from dust mites and microbes to activate PAR-2 and to enhance TSLP production [41,42,43]. Our study also revealed early development of a strong Th17 inflammatory axis in *Spink5* ^*-/-*^ skin which was totally blocked after *Klk5* knockout, supporting the role of Klk5 not only in desmosomal cleavage, but also in the development of Il-17 inflammation. In psoriasis and atopic dermatitis pathogeneses, Th17 cells play an important role in skin inflammation [44,45,46,47]. Il-17A has multiple effects and its main target cells in the skin are keratinocytes. This cytokine increases the expression of antimicrobial peptides, including members of the β-defensin and s100a families, thus stimulating the immune system [34,48]. In addition, IL-17A stimulates keratinocyte expression of multiple chemokines, including CCL20 which may directly recruit CCR6^+^ cells to the skin, including Th17 and dendritic cells, thereby establishing a chemotactic feedback loop for maintaining inflammatory cells in lesional skin [49]. Il-17A can also contribute to epidermal proliferation and skin barrier disruption. Uncovering of a strong Th17 inflammatory axis in *Spink5* ^*-/-*^ skin allows a better understanding of the disease and points to new therapeutic options for NS patients such as IL-17 inhibitors. The mechanisms by which Klk5 deletion totally blocks the development of Th17 response in *Spink5* ^*-/-*^ skin remain to be determined. Nonetheless, one could postulate that although IL-17 targeting has the potential to block skin inflammation in NS, *stratum corneum* detachment would probably not be prevented by this approach, in contrast to KLK5 inhibition which is likely to impact both pathological cascades.

In summary, in this study we have validated the efficacy of KLK5 knockout to reverse major skin abnormalities in NS leading to clinical, morphological and functional correction of the skin. These results establish a central role of KLK5 in NS symptoms and in the complex network of dysregulated cutaneous proteolytic activity. They revealed early development of a strong Th17 response in NS which was totally abolished by Klk5 deletion. Skin abnormalities in NS cause a major epidermal barrier deficiency leading to dehydration, severe cutaneous and systemic infections and inflammation. KLK5 inhibition has the potential to block these events in NS skin and thus appears as a major and promising target for drug development.

## Materials and Methods

### Materials

All chemicals were obtained from Sigma or Merck. Antibodies against Dsg1 (H-290), Par-2 (SAM11), Dsc1 (L-15), involucrin (M-15), and NIMP-R14 were obtained from Santa Cruz, Tslp (AF555) from R&D systems, loricrin (PRB-145) and filaggrin (PRB-147) from Covance.

### Animal handling

All experiments with animals were approved by local ethic committee CEEA 34 Paris Descartes and carried out according to our Institutions Guidelines and EU legislation.

### Generation of the *Klk5* ^*-/-*^

Mouse embryonic stem cells with targeted deletion in the *Klk5* gene were obtained from KOMP (http://www.komp.org) and used to derive chimeric mice with diploid aggregation chimeras [50]. Chimeric mice were found to be 100% transmitters and gave birth to *Klk5* ^*+/-*^ mice, which were intercrossed to obtain *Klk5* ^*-/-*^ mice.

### Genotyping

Genomic DNA was isolated from mouse-tails using Nucleospin (Macherey-Nagel) and subjected to PCR using GoTaq polymerase (Promega). Primer sequences are shown in S1 Table.

### RNA isolation and reverse transcription

Total RNAs from mouse tissues were extracted with RNeasy (Qiagen) and treated with DNase according to manufacturer’s instructions. The quality and quantity of RNA were determined by agarose electrophoresis and spectrophotometry. Reverse transcription was carried out with 1 μg of total RNA with MMLV reverse transcriptase (Invitrogen).

### Real-time PCR

cDNAs in 25 μl total volume were amplified with Mesa Green (Eurogentec). The sequences of gene-specific primers are given in S1 Table. Gene expression was normalized against *Hprt1*.

### Transepidermal water loss

At least three different measurements were taken for each mouse and averaged and at least eight mice from each genotype were used. Measurements were performed with the EP1 evaporimeter (ServoMed) as described [12]. Results were reported as fold increase over the wt control in order to exclude day-to-day variations in TEWL values depending on independent environmental conditions *e*.*g*. humidity.

### Toluidine blue staining of neonates

The method of polar lipid removal was used [51]. Neonates were euthanatized and dehydrated by sequential incubation in 25, 50 and 75% methanol in PBS (1 min per step) and finally in 100% methanol. Then, neonates were rehydrated by incubation in the same methanol solutions but in reverse order, washed with PBS and stained with 0.1% toluidine blue O in PBS for 1 h. Mice were photographed following destaining in PBS (2 washes of 1 and 10 min, respectively).

### Histology

Skin tissues were fixed in 4% formaldehyde in PBS pH 7.4 for 24 h and, then, embedded in paraffin. 5 μm sections were cut with a microtome. Hematoxylin/eosin/safranin (HES) and toluidine blue staining were performed on paraffin-embedded sections using standard histological techniques.

### Immunohistochemistry

Skin tissues were embedded in OCT and sectioned to 5 μm. The sections were fixed in acetone for 10 min, air-dried for 5 min, rehydrated with PBS for 5 min and the endogenous peroxidase activity was blocked with peroxidase blocking solution for 8 min at room temperature (Dako). The slides were incubated in PBS containing 0.3% BSA for blocking and 0.1% Triton X-100 for membrane permeabilisation for 5 min at room temperature. The antibody against, Tslp or Par2 was used at a 1:200 dilution and washed with PBS containing 0.3% BSA. Following incubation with the appropriate secondary antibody (Dako), slides were incubated with the chromogen solution (Dako).

### Immunofluorescent stainings

For immunofluorescent stainings, 5 μm paraffin sections were cut from the biopsies, deparaffinized, rehydrated and followed by antigen retrieval using sodium citrate buffer (pH 6). After a 30 minute incubation period with 3% BSA in PBS, the primary antibody was incubated overnight at 4°C at 1:1000 for Dsc1, Dsg1 and NIMPr14 and 1:500 for involucrin, loricrin and filaggrin. The following day, the appropriate secondary antibody was incubated for 60 minutes at room temperature. Nuclei were staining using DAPI at 1μg/ml. Images were taken using a Leica TCS SP8 SMD confocal microscope. Data were analyzed using ImageJ.

### Scanning and transmission electron microscopy

For SEM, neonates were decapitated and their heads were fixed in 4% formaldehyde in PBS for 24 h, then, washed twice with PBS and dehydrated in a series of ethanol solutions 25, 50, and 100% (10 min each). Finally, ethanol was replaced with 100% acetone (2 washes, 10 min each). The samples were dried, covered with gold and observed in a Field-Emission Scanning Electron Microscope (JEOL, 6300). For TEM, skin from neonates was excised with 4 mm skin biopsy punches and fixed in a 2% glutaraldehyde and 4% formaldehyde solution in PBS pH 7.4 at 4°C. Then, samples were processed and stained as described [12].

### *In situ* zymography

Skin cryosections (5 μm thick) were mounted on glass slides, rinsed with 2% Tween 20 in PBS and incubated overnight at 37°C with 10 μg ml^-1^ BODIPY FL casein (Life Technologies) or 100 μg ml^-1^ BODIPY FL elastin (Life Technologies) in 50 mM Tris-HCl, pH 8.0. Sections were rinsed with PBS and visualized with a Leica TCS SP5 AOBS confocal laser scanning microscope (CLSM). Data were analyzed using ImageJ.

### Proteolytic activity assays

For proteolytic activity assays, skin was crushed in 1 M acetic acid using a Fast Prep (MP Biomedicals). After overnight extraction at 4°C, insoluble material was removed by centrifugation (13,000 g, 4°C for 30 min) and the supernatant was dried using a Speed-Vac. Proteins were resuspended in water overnight at 4°C and clarified by centrifugation (13,000 g, 4°C for 30 min). Protein content was determined by Bradford assay (Bio-Rad Laboratories). Proteolytic activity was assessed using colorimetric peptide substrates that are preferentially cleaved by different proteases. 25μg of proteins were added to assay buffer (0.1 M Tris-HCl pH 8.0, 0.005% Triton X-100, 0.05% sodium azide) in 96-well plates (final volume 200 μl). Substrates used were 150 μM KHLY-pNA (cleaved by KLK7) and 150 μM Ac-WAVR-pNA (cleaved by KLK14; [24,25]). Plates were incubated at 37°C overnight and activity was analysed by measuring the increase in absorbance at 405 nm compared with substrate only controls.

### Filipin staining

Before staining cryosections were washed with PBS for 5 min and incubated with filipin (diluted with PBS to 50 μg/ml) for 30 min at room temperature and in the dark, then, washed with PBS for 10 min mounted with aqueous medium (Dako) and visualized with CLSM with excitation and emission wavelengths 405 and 480 nm, respectively.

### Nile red

Nile red was dissolved in acetone and stored at -20°C and, prior to use, diluted to 5 μg/ml in 75% glycerol in water. A drop of this solution was placed on each section and visualized with CLSM with excitation at 488 nm and emission at 520 and 600 nm.

### Statistical analysis

All data were analyzed using GraphPad Prism v5 software. In all experiments, at least 4 independent animals per genotype were used. Experiments were repeated two to three times.

In figures, the results of one representative experiment are shown as mean values ± SEM. Comparison between values was performed using the non-parametric Mann-Whitney U-test. P values < 0.05 were considered statistically significant.

## Supporting Information

S1 Fig Generation of *Klk5* ^*-/-*^ mice. (**A**) Schematic representation of the knockout cassette between exons 1 and 3 of *Klk5* and annealing positions of primers used for genotyping. (**B**) Example of genomic PCR for wt, heterozygous (*Klk5* ^*+/-*^) and knockout (*Klk5* ^*-/-*^) alleles. (**C**) Tissue expression of the *Klk5* gene by RT-PCR, demonstrating strong expression in mouse skin. Complete ablation of *Klk5* in expressing tissues was confirmed in *Klk5* ^*-/-*^ mice by semi-quantitative RT-PCR (**D**) and in skin (**E**) by RT-qPCR.
(TIF)

S2 Fig Expression of *Klk5* and *Spink5* mRNA in murine skin. Absence of detectable levels of *Klk5* and *Spink5* mRNA after *Klk5* and *Spink5* knockout in skin. wt and *Spink5-/-* animals express *Klk5* whereas *Spink5 is* expressed in wt and *Klk5* ^*-/-*^ mice only. Absence of both mRNA is confirmed in *Spink5* ^*-/-*^ */Klk5* ^*-/-*^. Data are shown as the mean ± s.e.m. of triplicate amplification for at least four mice per genotype. Results are normalized to wt mean (set as 1.0).
(TIF)

S3 Fig Quantification of corneodesmosomes in the *stratum corneum*. Corneodesmosomes were quantified on TEM pictures between 2 layers of corneocytes. (**A**) Total number of corneodesmosomes (uncleaved and split ones) is higher in *Klk5* ^*-/-*^ and *Spink5* ^*-/-*^ */Klk5* ^*-/-*^; (**B**) *Klk5* ^*-/-*^ and *Spink5* ^*-/-*^ */Klk5* ^*-/-*^ show an increased number of uncleaved corneodesmosomes. Data are shown as the mean ± s.e.m. At least 140 corneodesmosomes were counted for each genotype, *p<0.05, **p<0.005, ***p<0.001 (Mann-Whitney U test).
(TIF)

S4 Fig *Spink5* ^*-/-*^ *Klk5* ^*-/-*^ skin does not show evidence of abnormal epidermal differentiation. (**A**) Deletion of Klk5 blocks excessive profilaggrin processing in Lekti-deficient epidermis. Immunodetection of profilaggrin (proFlg), filaggrin (Flg), and filaggrin-processing intermediate dimers (2xFlg) and trimers (3xFlg) in epidermal extracts from newborn wt, *Spink5* ^*-/-*^ and *Spink5* ^*-/-*^ *Klk5* ^*-/-*^ detects no abnormal Flg processing in *Spink5* ^*-/-*^ *Klk5* ^*-/-*^ epidermis. To analyze epidermal lipid organization, skin cryosections were stained with filipin (**B**) to visualize free cholesterol, and with Nile red (**C**) for neutral and polar lipids.
(TIF)

S5 Fig *Klk5* deletion results in suppression of the cutaneous inflammatory phenotype of NS. **(A)** Par2 immunostaining shows normalized Par2 expression in *Spink5* ^*-/-*^ *Klk5* ^*-/-*^ compared to *Spink5* ^*-/-*^; scale bar: 50μm (**B-E**). Analysis of mRNA expression of cytokines and chemokines in newborn skin quantified by RT-qPCR. (**B**) Il-25 alarmin; (**C**) Ccl17 (Tarc), Ccl22 (Mdc), Ccl8; (**D**) Il-13; (**E**) Th1 regulated genes Ccl5, Cxcl9, Cxcl10 and Cxcl11. Data are shown as the mean ± s.e.m. of triplicate amplification for at least five mice per genotype. Results are normalized to wt mean (set as 1.0). *p<0.05, **p<0.005, ***p<0.001 (Mann-Whitney U test). (**F**) Immunohistological analysis of neutrophils (white arrows) in newborn skin shows important neutrophil infiltrate in *Spink5* ^*-/-*^ dermis and rare neutrophils in skin of the other genotypes, nuclei are in blue and neutrophils stained in red; scale bar: 25μm.
(TIF)

S6 Fig Control stainings with isotypes and secondary antibodies. **(A)** Staining with isotype and anti-rabbit AF488 secondary antibody, control for Dsg1, Flg and Loricrin immunostainings; scale bar: 25μm **(B)** Staining with isotype and anti-goat AF555 secondary antibody, control for Dsc1 and Involucrin stainings; scale bar: 25μm **(C)** Staining with isotype and anti-mouse-HRP secondary antibody, control for Par-2 staining; scale bar: 50μm **(D)** Staining with isotype and anti-rabbit-HRP secondary antibody, control for Tslp staining; scale bar: 50μm **(E)** Staining with isotype and anti-rat AF555 secondary antibody, control for NIMP-r14 staining; scale bar: 25μm.
(TIF)

S7 Fig *Spink5* ^*-/-*^ *Klk5* ^*-/-*^ mice maintain an intact skin barrier with no sign of aberrant desquamation or inflammation. Skin barrier integrity and function is drastically improved in the *Spink5* ^*-/-*^ *Klk5* ^*-/-*^ as assessed by the toluidine blue dye penetration assay. Only a few patches of dye penetration are observed on the body of *Spink5* ^*-/-*^ *Klk5* ^*-/-*^ mice two days after birth.
(TIF)

S1 Table Primer sequences and conditions used for genotyping, real-time PCR, and semi-quantitative RT-PCR. (PDF)

## References

[1] Simpson CL, Patel DM, Green KJ (2011) Deconstructing the skin: cytoarchitectural determinants of epidermal morphogenesis. Nat Rev Mol Cell Biol 12: 565–580. 10.1038/nrm3175 21860392 PMC3280198

[2] Fuchs E (2007) Scratching the surface of skin development. Nature 445: 834–842. 17314969 10.1038/nature05659 PMC2405926

[3] Netherton EW (1958) "A unique case of trichorrhexis nodosa: bamboo hairs. AMA Arch Derm 78: 483–487. 13582191 10.1001/archderm.1958.01560100059009

[4] Comel M (1949) Ichthyosis Linearis circumflexa. Dermatologica 98: 133–136. 18152084

[5] Hovnanian A (2012) Netherton syndrome: new advances in clinic, disease mechanism and treatment. Expert review 7: 81–92.

[6] Ong C, Harper J (2006) Netherton's syndrome In: Harper J, Orange A, Prose N, editors. Textbook of pediatric Dermatology. Second ed. Turin, Italy: Blackwell pp. 1359–1366.

[7] Chavanas S, Bodemer C, Rochat A, Hamel-Teillac D, Ali M, et al (2000) Mutations in SPINK5, encoding a serine protease inhibitor, cause Netherton syndrome. Nat Genet 25: 141–142. 10835624 10.1038/75977

[8] Deraison C, Bonnart C, Lopez F, Besson C, Robinson R, et al (2007) LEKTI fragments specifically inhibit KLK5, KLK7, and KLK14 and control desquamation through a pH-dependent interaction. Mol Biol Cell 18: 3607–3619. 17596512 10.1091/mbc.E07-02-0124 PMC1951746

[9] Fortugno P, Bresciani A, Paolini C, Pazzagli C, El Hachem M, et al (2011) Proteolytic activation cascade of the Netherton syndrome-defective protein, LEKTI, in the epidermis: implications for skin homeostasis. J Invest Dermatol 131: 2223–2232. 10.1038/jid.2011.174 21697885

[10] Egelrud T, Brattsand M, Kreutzmann P, Walden M, Vitzithum K, et al (2005) hK5 and hK7, two serine proteinases abundant in human skin, are inhibited by LEKTI domain 6. Br J Dermatol 153: 1200–1203. 16307658 10.1111/j.1365-2133.2005.06834.x

[11] Borgoño CA, Michael IP, Komatsu N, Jayakumar A, Kapadia R, et al (2007) A potential role for multiple tissue kallikrein serine proteases in epidermal desquamation. J Biol Chem 282: 3640–3652. 17158887 10.1074/jbc.M607567200

[12] Descargues P, Deraison C, Bonnart C, Kreft M, Kishibe M, et al (2005) Spink5-deficient mice mimic Netherton syndrome through degradation of desmoglein 1 by epidermal protease hyperactivity. Nat Genet 37: 56–65. 15619623 10.1038/ng1493

[13] Hewett DR, Simons AL, Mangan NE, Jolin HE, Green SM, et al (2005) Lethal, neonatal ichthyosis with increased proteolytic processing of filaggrin in a mouse model of Netherton syndrome. Hum Mol Genet 14: 335–346. 15590704 10.1093/hmg/ddi030

[14] Yang T, Liang D, Koch PJ, Hohl D, Kheradmand F, et al (2004) Epidermal detachment, desmosomal dissociation, and destabilization of corneodesmosin in Spink5-/- mice. Genes Dev 18: 2354–2358. 15466487 10.1101/gad.1232104 PMC522985

[15] Briot A, Deraison C, Lacroix M, Bonnart C, Robin A, et al (2009) Kallikrein 5 induces atopic dermatitis-like lesions through PAR2-mediated thymic stromal lymphopoietin expression in Netherton syndrome. J Exp Med 206: 1135–1147. 10.1084/jem.20082242 19414552 PMC2715042

[16] Briot A, Lacroix M, Robin A, Steinhoff M, Deraison C, et al (2010) Par2 inactivation inhibits early production of TSLP, but not cutaneous inflammation, in Netherton syndrome adult mouse model. J Invest Dermatol 130: 2736–2742. 10.1038/jid.2010.233 20703245

[17] Caubet C, Jonca N, Brattsand M, Guerrin M, Bernard D, et al (2004) Degradation of corneodesmosome proteins by two serine proteases of the kallikrein family, SCTE/KLK5/hK5 and SCCE/KLK7/hK7. J Invest Dermatol 122: 1235–1244. 15140227 10.1111/j.0022-202X.2004.22512.x

[18] Ovaere P, Lippens S, Vandenabeele P, Declercq W (2009) The emerging roles of serine protease cascades in the epidermis. Trends Biochem Sci 34: 453–463. 10.1016/j.tibs.2009.08.001 19726197

[19] Miyai M, Matsumoto Y, Yamanishi H, Yamamoto-Tanaka M, Tsuboi R, et al (2014) Keratinocyte-Specific Mesotrypsin Contributes to the Desquamation Process via Kallikrein Activation and LEKTI Degradation. J Invest Dermatol. 10.1038/jid.2014.3 24390132

[20] Sales KU, Masedunskas A, Bey AL, Rasmussen AL, Weigert R, et al (2010) Matriptase initiates activation of epidermal pro-kallikrein and disease onset in a mouse model of Netherton syndrome. Nat Genet 42: 676–683. 10.1038/ng.629 20657595 PMC3081165

[21] de Veer SJ, Furio L, Harris JM, Hovnanian A (2014) Proteases and proteomics: cutting to the core of human skin pathologies. Proteomics Clin Appl 8: 389–402. 10.1002/prca.201300081 24677727

[22] Bonnart C, Deraison C, Lacroix M, Uchida Y, Besson C, et al (2010) Elastase 2 is expressed in human and mouse epidermis and impairs skin barrier function in Netherton syndrome through filaggrin and lipid misprocessing. J Clin Invest 120: 871–882. 10.1172/JCI41440 20179351 PMC2827963

[23] Furio L, de Veer S, Jaillet M, Briot A, Robin A, et al (2014) Transgenic kallikrein 5 mice reproduce major cutaneous and systemic hallmarks of Netherton syndrome. J Exp Med 211: 499–513. 10.1084/jem.20131797 24534191 PMC3949577

[24] de Veer SJ, Swedberg JE, Parker EA, Harris JM (2012) Non-combinatorial library screening reveals subsite cooperativity and identifies new high-efficiency substrates for kallikrein-related peptidase 14. Biol Chem 393: 331–341. 10.1515/bc-2011-250 22505516

[25] de Veer SJ, Ukolova SS, Munro CA, Swedberg JE, Buckle AM, et al (2013) Mechanism-based selection of a potent kallikrein-related peptidase 7 inhibitor from a versatile library based on the sunflower trypsin inhibitor SFTI-1. Biopolymers 100: 510–518. 10.1002/bip.22231 24078181

[26] Brattsand M, Egelrud T (1999) Purification, molecular cloning, and expression of a human stratum corneum trypsin-like serine protease with possible function in desquamation. J Biol Chem 274: 30033–30040. 10514489 10.1074/jbc.274.42.30033

[27] Suzuki Y, Nomura J, Hori J, Koyama J, Takahashi M, et al (1993) Detection and characterization of endogenous protease associated with desquamation of stratum corneum. Arch Dermatol Res 285: 372–377. 8215586 10.1007/BF00371839

[28] Descargues P, Deraison C, Prost C, Fraitag S, Mazereeuw-Hautier J, et al (2006) Corneodesmosomal cadherins are preferential targets of stratum corneum trypsin- and chymotrypsin-like hyperactivity in Netherton syndrome. J Invest Dermatol 126: 1622–1632. 16628198 10.1038/sj.jid.5700284

[29] Fartasch M, Williams ML, Elias PM (1999) Altered lamellar body secretion and stratum corneum membrane structure in Netherton syndrome: differentiation from other infantile erythrodermas and pathogenic implications. Arch Dermatol 135: 823–832. 10411158 10.1001/archderm.135.7.823

[30] Hausser I, Anton-Lamprecht I (1996) Severe congenital generalized exfoliative erythroderma in newborns and infants: a possible sign of Netherton syndrome. Pediatr Dermatol 13: 183–199. 8806118 10.1111/j.1525-1470.1996.tb01202.x

[31] Yamasaki K, Schauber J, Coda A, Lin H, Dorschner RA, et al (2006) Kallikrein-mediated proteolysis regulates the antimicrobial effects of cathelicidins in skin. Faseb J 20: 2068–2080. 17012259 10.1096/fj.06-6075com

[32] Yamasaki K, Di Nardo A, Bardan A, Murakami M, Ohtake T, et al (2007) Increased serine protease activity and cathelicidin promotes skin inflammation in rosacea. Nat Med 13: 975–980. 17676051 10.1038/nm1616

[33] Homey B, Zlotnik A (1999) Chemokines in allergy. Curr Opin Immunol 11: 626–634. 10631546 10.1016/s0952-7915(99)00028-x

[34] Nograles KE, Zaba LC, Guttman-Yassky E, Fuentes-Duculan J, Suarez-Farinas M, et al (2008) Th17 cytokines interleukin (IL)-17 and IL-22 modulate distinct inflammatory and keratinocyte-response pathways. Br J Dermatol 159: 1092–1102. 10.1111/j.1365-2133.2008.08769.x 18684158 PMC2724264

[35] Boniface K, Bernard FX, Garcia M, Gurney AL, Lecron JC, et al (2005) IL-22 inhibits epidermal differentiation and induces proinflammatory gene expression and migration of human keratinocytes. J Immunol 174: 3695–3702. 15749908 10.4049/jimmunol.174.6.3695

[36] Fischer J, Meyer-Hoffert U (2013) Regulation of kallikrein-related peptidases in the skin—from physiology to diseases to therapeutic options. Thromb Haemost 110: 442–449. 10.1160/TH12-11-0836 23446429

[37] Sotiropoulou G, Pampalakis G (2010) Kallikrein-related peptidases: bridges between immune functions and extracellular matrix degradation. Biol Chem 391: 321–331. 10.1515/BC.2010.036 20180637

[38] Sakabe J, Yamamoto M, Hirakawa S, Motoyama A, Ohta I, et al (2013) Kallikrein-related peptidase 5 functions in proteolytic processing of profilaggrin in cultured human keratinocytes. J Biol Chem 288: 17179–17189. 10.1074/jbc.M113.476820 23629652 PMC3682523

[39] Soumelis V, Reche PA, Kanzler H, Yuan W, Edward G, et al (2002) Human epithelial cells trigger dendritic cell mediated allergic inflammation by producing TSLP. Nat Immunol 3: 673–680. 12055625 10.1038/ni805

[40] Sun JD, Linden KG (2006) Netherton syndrome: a case report and review of the literature. Int J Dermatol 45: 693–697. 16796630 10.1111/j.1365-4632.2005.02637.x

[41] Cork MJ, Danby SG, Vasilopoulos Y, Hadgraft J, Lane ME, et al (2009) Epidermal barrier dysfunction in atopic dermatitis. J Invest Dermatol 129: 1892–1908. 10.1038/jid.2009.133 19494826

[42] Takai T, Ikeda S (2011) Barrier dysfunction caused by environmental proteases in the pathogenesis of allergic diseases. Allergol Int 60: 25–35. 10.2332/allergolint.10-RAI-0273 21173566

[43] Angelova-Fischer I, Fernandez IM, Donnadieu MH, Bulfone-Paus S, Zillikens D, et al (2010) Injury to the stratum corneum induces in vivo expression of human thymic stromal lymphopoietin in the epidermis. J Invest Dermatol 130: 2505–2507. 10.1038/jid.2010.143 20555350

[44] Lynde CW, Poulin Y, Vender R, Bourcier M, Khalil S (2014) Interleukin 17A: toward a new understanding of psoriasis pathogenesis. J Am Acad Dermatol 71: 141–150. 10.1016/j.jaad.2013.12.036 24655820

[45] Guttman-Yassky E, Dhingra N, Leung DY (2013) New era of biologic therapeutics in atopic dermatitis. Expert Opin Biol Ther 13: 549–561. 10.1517/14712598.2013.758708 23323893 PMC3819721

[46] Suarez-Farinas M, Dhingra N, Gittler J, Shemer A, Cardinale I, et al (2013) Intrinsic atopic dermatitis shows similar TH2 and higher TH17 immune activation compared with extrinsic atopic dermatitis. J Allergy Clin Immunol 132: 361–370. 10.1016/j.jaci.2013.04.046 23777851 PMC3991240

[47] Koga C, Kabashima K, Shiraishi N, Kobayashi M, Tokura Y (2008) Possible pathogenic role of Th17 cells for atopic dermatitis. J Invest Dermatol 128: 2625–2630. 10.1038/jid.2008.111 18432274

[48] Liang SC, Tan XY, Luxenberg DP, Karim R, Dunussi-Joannopoulos K, et al (2006) Interleukin (IL)-22 and IL-17 are coexpressed by Th17 cells and cooperatively enhance expression of antimicrobial peptides. J Exp Med 203: 2271–2279. 16982811 10.1084/jem.20061308 PMC2118116

[49] Harper EG, Guo C, Rizzo H, Lillis JV, Kurtz SE, et al (2009) Th17 cytokines stimulate CCL20 expression in keratinocytes in vitro and in vivo: implications for psoriasis pathogenesis. J Invest Dermatol 129: 2175–2183. 10.1038/jid.2009.65 19295614 PMC2892172

[50] Tanaka M, Hadjantonakis AK, Vintersten K, Nagy A (2009) Aggregation chimeras: combining ES cells, diploid, and tetraploid embryos. Methods Mol Biol 530: 287–309. 10.1007/978-1-59745-471-1_15 19266342 PMC2913464

[51] Hardman MJ, Sisi P, Banbury DN, Byrne C (1998) Patterned acquisition of skin barrier function during development. Development 125: 1541–1552. 9502735 10.1242/dev.125.8.1541

