# Supplementary figures and images for: KLK5 Inactivation Reverses Cutaneous Hallmarks of Netherton Syndrome
Source: PLoS Genet. 2015 Sep 21;11(9):e1005389. doi: 10.1371/journal.pgen.1005389 (PMC4577096; doi:10.1371/journal.pgen.1005389)

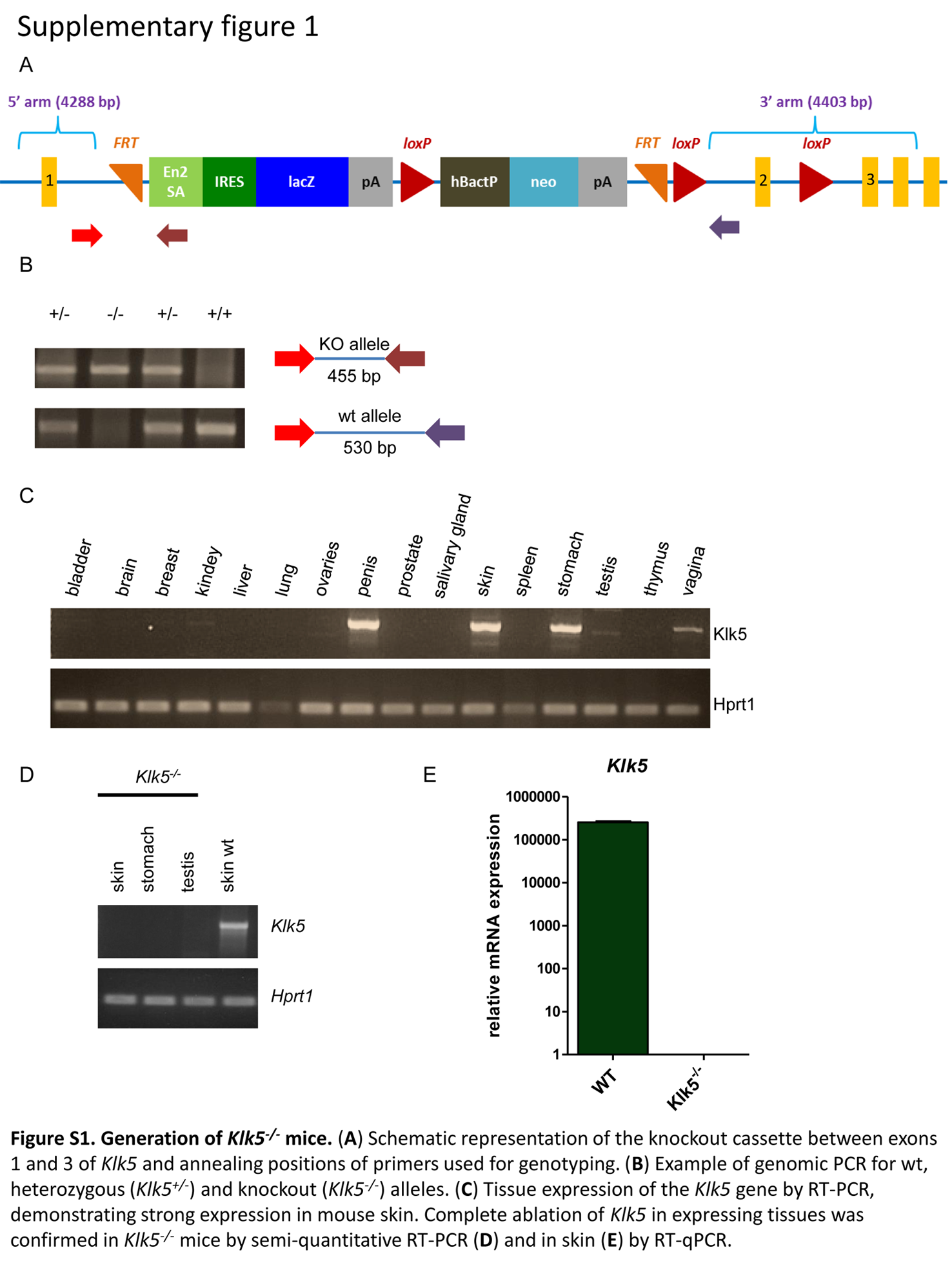

Supplement: S1 Fig — (A) Schematic representation of the knockout cassette between exons 1 and 3 of Klk5 and annealing positions of primers used for genotyping. (B) Example of genomic PCR for wt, heterozygous (Klk5 +/-) and knockout (Klk5 -/-) alleles. (C) Tissue expression of the Klk5 gene by RT-PCR, demonstrating strong expression in mouse skin. Complete ablation of Klk5 in expressing tissues was confirmed in Klk5 -/- mice by semi-quantitative RT-PCR (D) and in skin (E) by RT-qPCR. (TIF) [file pgen.1005389.s001.tif]

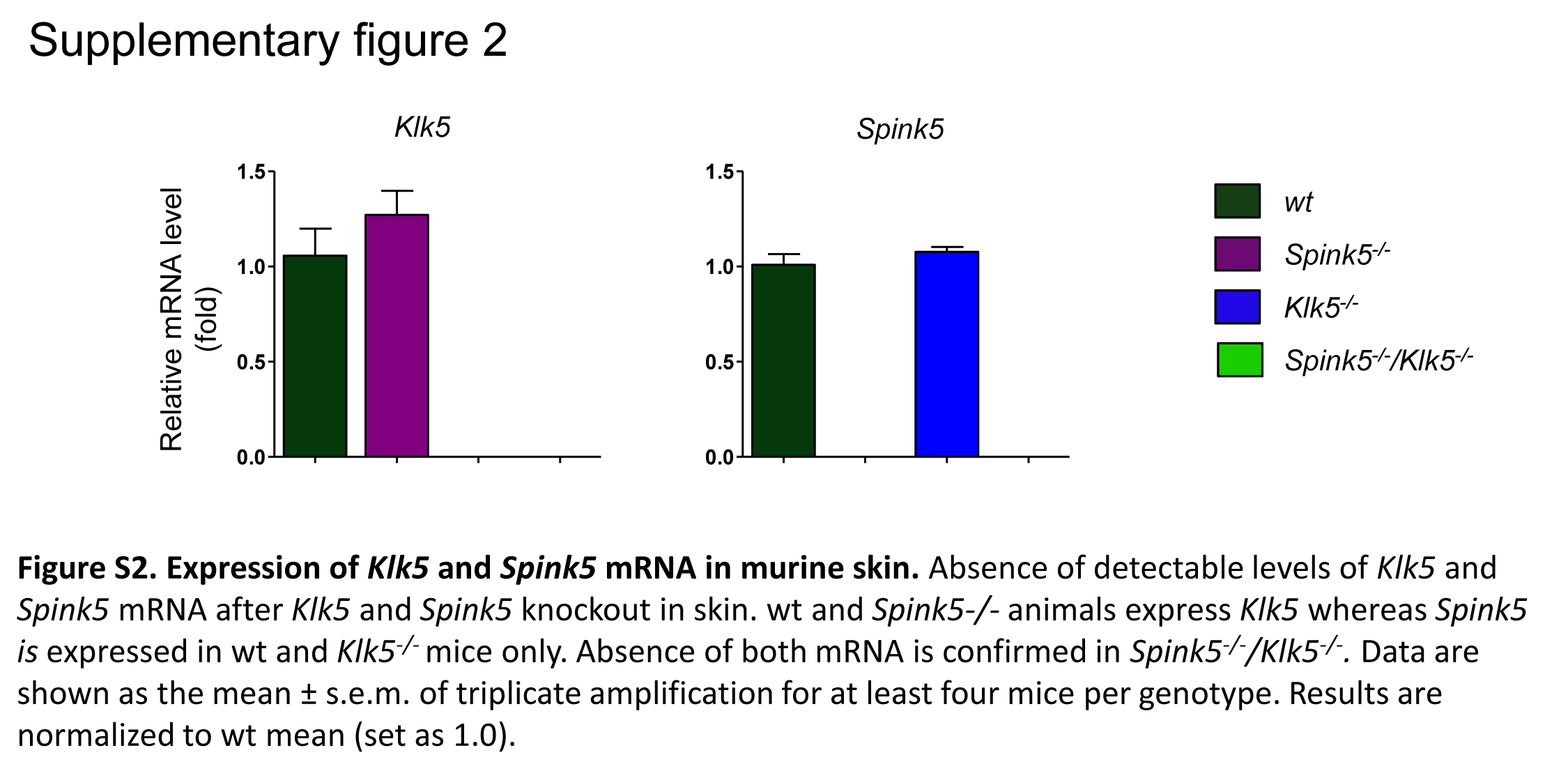

Supplement: S2 Fig — Absence of detectable levels of Klk5 and Spink5 mRNA after Klk5 and Spink5 knockout in skin. wt and Spink5-/- animals express Klk5 whereas Spink5 is expressed in wt and Klk5 -/- mice only. Absence of both mRNA is confirmed in Spink5 -/- /Klk5 -/-. Data are shown as the mean ± s.e.m. of triplicate amplification for at least four mice per genotype. Results are normalized to wt mean (set as 1.0). (TIF) [file pgen.1005389.s002.tif]

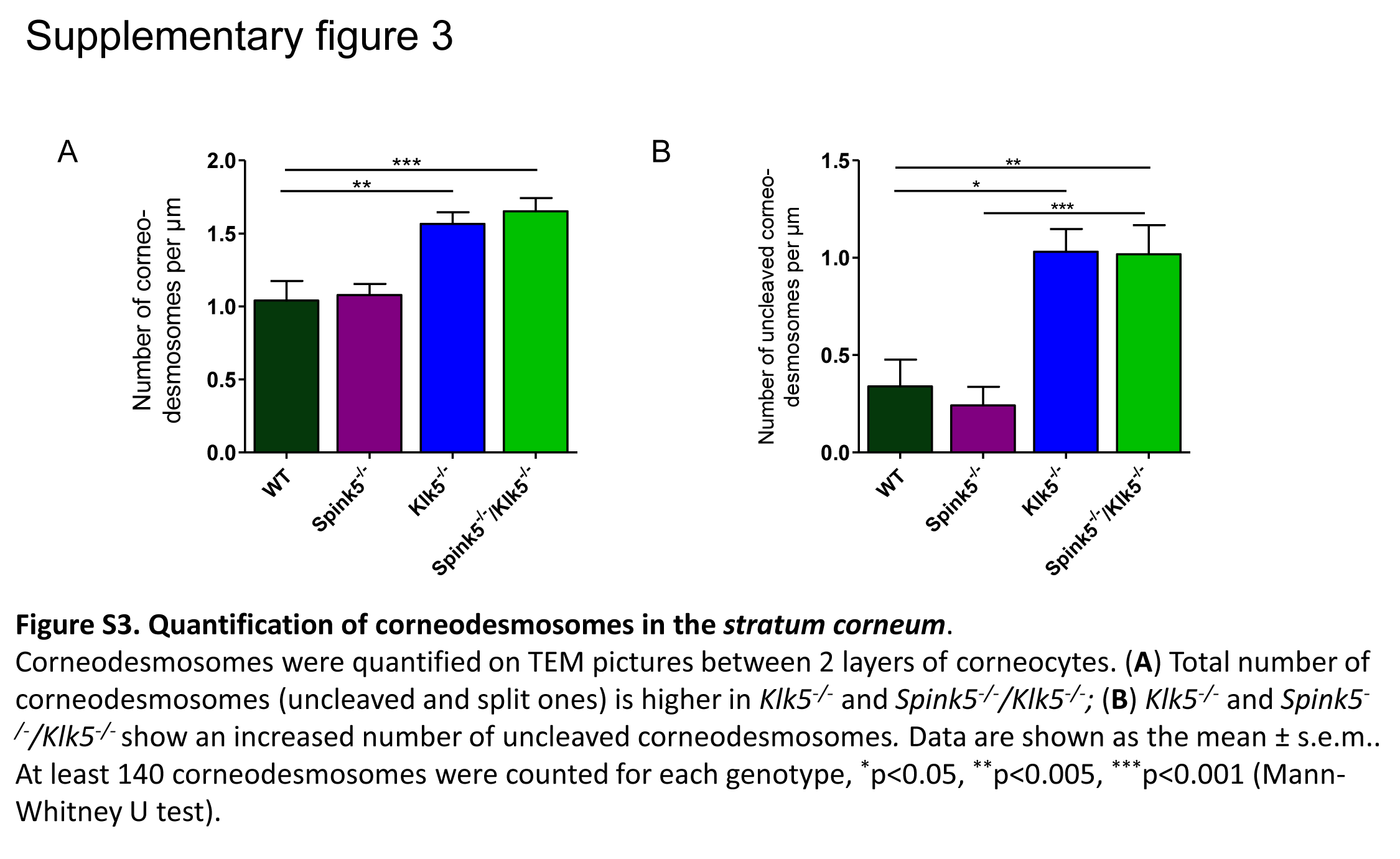

Supplement: S3 Fig — Corneodesmosomes were quantified on TEM pictures between 2 layers of corneocytes. (A) Total number of corneodesmosomes (uncleaved and split ones) is higher in Klk5 -/- and Spink5 -/- /Klk5 -/-; (B) Klk5 -/- and Spink5 -/- /Klk5 -/- show an increased number of uncleaved corneodesmosomes. Data are shown as the mean ± s.e.m. At least 140 corneodesmosomes were counted for each genotype, *p<0.05, **p<0.005, ***p<0.001 (Mann-Whitney U test). (TIF) [file pgen.1005389.s003.tif]

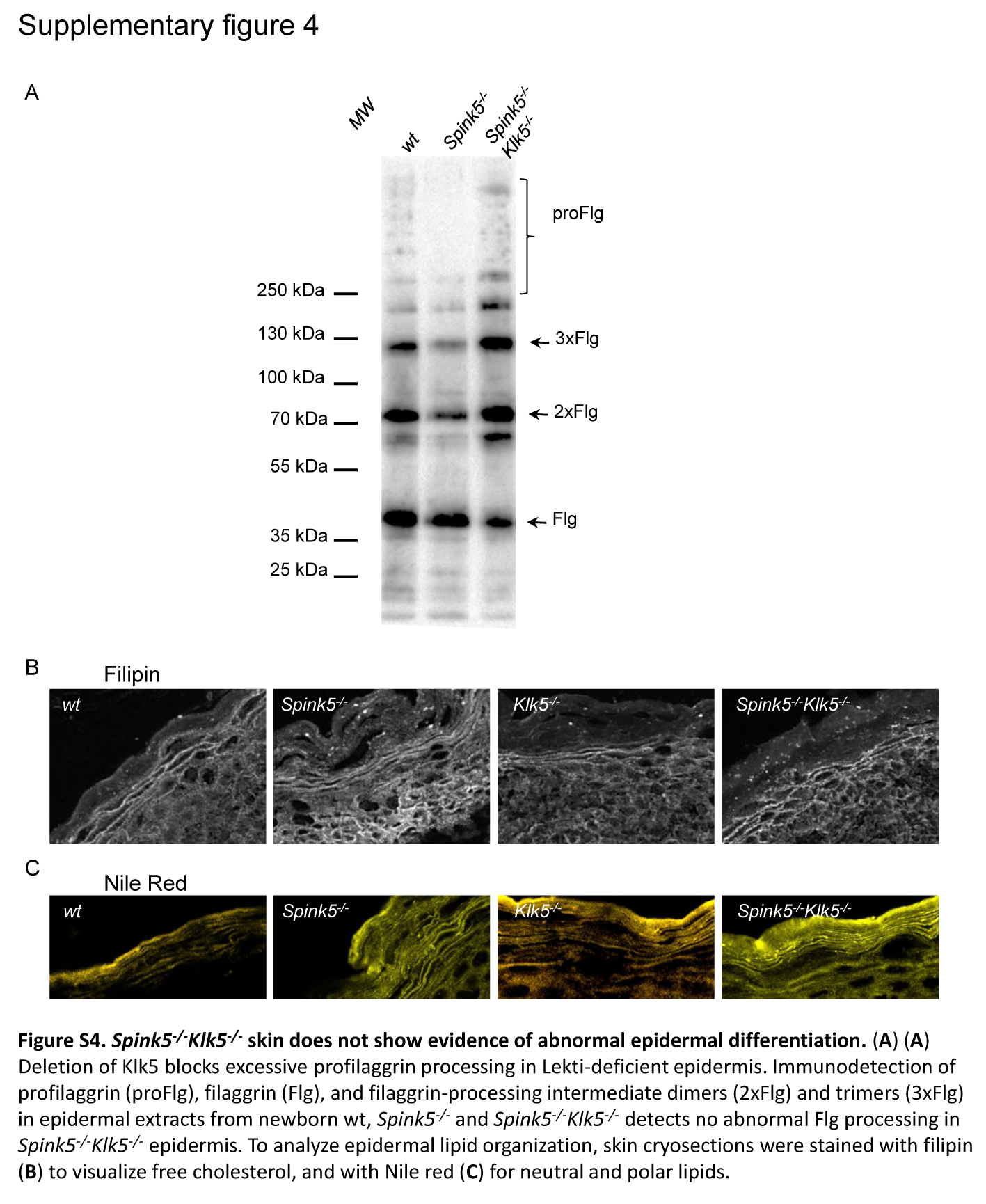

Supplement: S4 Fig — (A) Deletion of Klk5 blocks excessive profilaggrin processing in Lekti-deficient epidermis. Immunodetection of profilaggrin (proFlg), filaggrin (Flg), and filaggrin-processing intermediate dimers (2xFlg) and trimers (3xFlg) in epidermal extracts from newborn wt, Spink5 -/- and Spink5 -/- Klk5 -/- detects no abnormal Flg processing in Spink5 -/- Klk5 -/- epidermis. To analyze epidermal lipid organization, skin cryosections were stained with filipin (B) to visualize free cholesterol, and with Nile red (C) for neutral and polar lipids. (TIF) [file pgen.1005389.s004.tif]

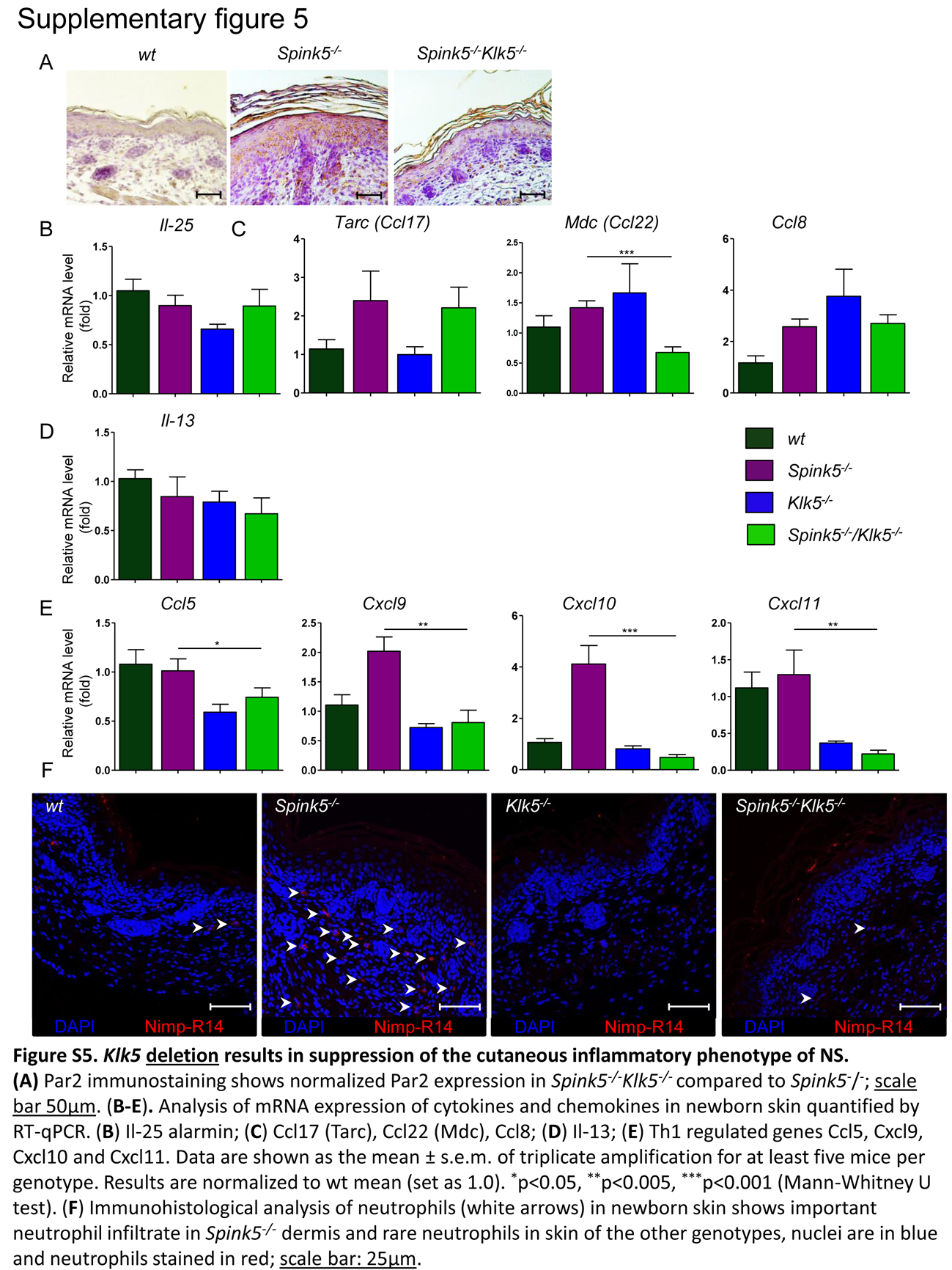

Supplement: S5 Fig — (A) Par2 immunostaining shows normalized Par2 expression in Spink5 -/- Klk5 -/- compared to Spink5 -/-; scale bar: 50μm (B-E). Analysis of mRNA expression of cytokines and chemokines in newborn skin quantified by RT-qPCR. (B) Il-25 alarmin; (C) Ccl17 (Tarc), Ccl22 (Mdc), Ccl8; (D) Il-13; (E) Th1 regulated genes Ccl5, Cxcl9, Cxcl10 and Cxcl11. Data are shown as the mean ± s.e.m. of triplicate amplification for at least five mice per genotype. Results are normalized to wt mean (set as 1.0). *p<0.05, **p<0.005, ***p<0.001 (Mann-Whitney U test). (F) Immunohistological analysis of neutrophils (white arrows) in newborn skin shows important neutrophil infiltrate in Spink5 -/- dermis and rare neutrophils in skin of the other genotypes, nuclei are in blue and neutrophils stained in red; scale bar: 25μm. (TIF) [file pgen.1005389.s005.tif]

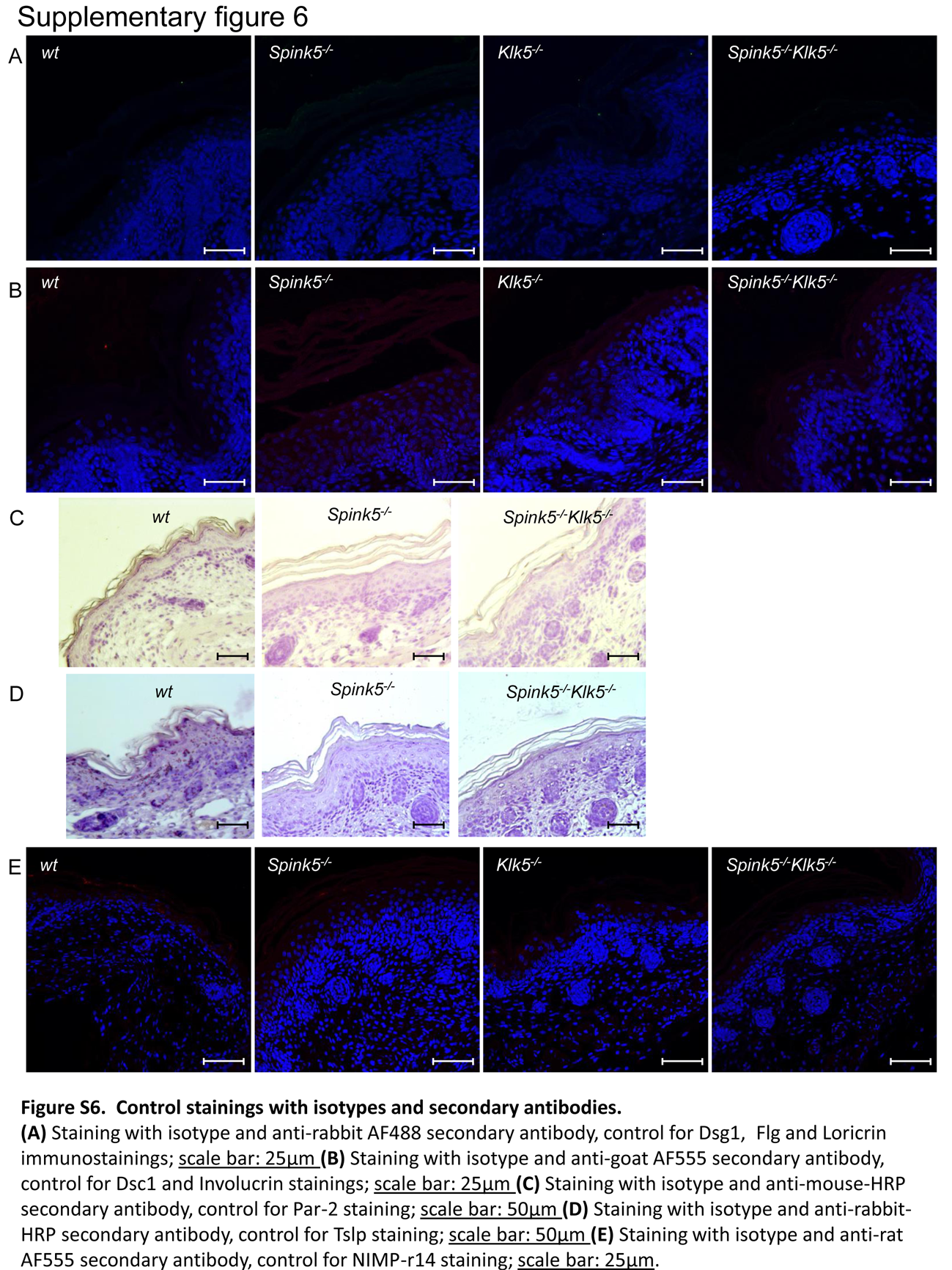

Supplement: S6 Fig — (A) Staining with isotype and anti-rabbit AF488 secondary antibody, control for Dsg1, Flg and Loricrin immunostainings; scale bar: 25μm (B) Staining with isotype and anti-goat AF555 secondary antibody, control for Dsc1 and Involucrin stainings; scale bar: 25μm (C) Staining with isotype and anti-mouse-HRP secondary antibody, control for Par-2 staining; scale bar: 50μm (D) Staining with isotype and anti-rabbit-HRP secondary antibody, control for Tslp staining; scale bar: 50μm (E) Staining with isotype and anti-rat AF555 secondary antibody, control for NIMP-r14 staining; scale bar: 25μm. (TIF) [file pgen.1005389.s006.tif]

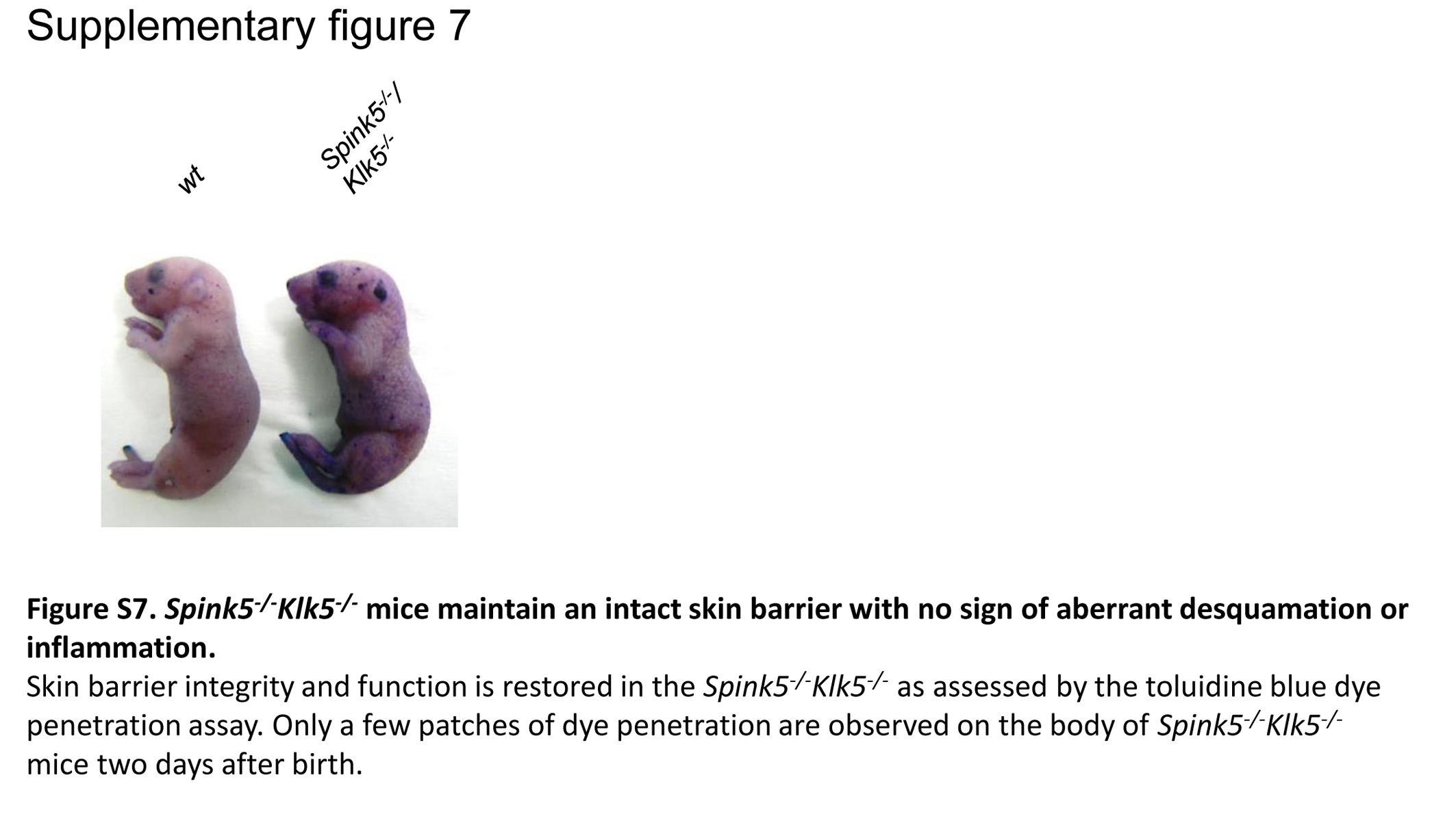

Supplement: S7 Fig — Skin barrier integrity and function is drastically improved in the Spink5 -/- Klk5 -/- as assessed by the toluidine blue dye penetration assay. Only a few patches of dye penetration are observed on the body of Spink5 -/- Klk5 -/- mice two days after birth. (TIF) [file pgen.1005389.s007.tif]
