# Supplementary material for: KLK5 Inactivation Reverses Cutaneous Hallmarks of Netherton Syndrome
Source: PLoS Genet. 2015 Sep 21;11(9):e1005389. doi: 10.1371/journal.pgen.1005389 (PMC4577096; doi:10.1371/journal.pgen.1005389)
Supplement: S1 Table — (PDF) [file pgen.1005389.s008.pdf]

Supplementary table 1

Gene	Forward primer	Reverse primer
Hprt	CTGGTTAAGCAGTACAGCCCCAA	CGAGAGGTCCTTTTCACCAGC
Tslp	CGACAGCATGGTTCTTCTCA	CGATTTGCTCGAACTTAGCC
Tnf-α	CCCCAAAGGGATGAGAAGTT	CACTTGGTGGTTTGCTACGA
Il-1β	GGGCCTCAAAGGAAAGAATC	TACCAGTTGGGGAACTCTGC
Il-4	GAAGAACACCACAGAGAGTGAGC	GGAAGTCTTTCAGTGATGTGGAC
Il-6	TTCCATCCAGTTGCCTTCTT	CATCATTTCTTTGTATCTCT
Il-13	ATTGCATGGCCTCTGTAACC	TGAGTCCACAGCTGAGATGC
Il-17a	GCTCCAGAAGGCCCTCAGA	AGCTTTCCCTCCGCATTGA
Il-18	ACAACTTTGGCCGACTTCAC	GGGTTCACTGGCACTTTGAT
Il-22	CAACTTCCAGCAGCCATACA	GTTGAGCACCTGCTTCATCA
Il23-p19	AATAATGTGCCCCGTATCCA	CTGGAGGAGTTGGCTGAGTC
Il-25	AGAACCCCCGGAGGAGTGGC	GTGGGCACAGGCATCGAGCG
Il-33	ATGGGAAGAAGCTGATGGTG	CCGAGGACTTTTTGTGAAGG
Ifn-γ	GCGTCATTGAATCACACCTG	TGAGCTCATTGAATGCTTGG
defb4	ACATTTCTCCTGGTGCTGCT	TCTGACTTTAAAATGGCCACAA
slpi	CACAATGCCGTACTGACTGG	GACATTGGGAGGGTTAAGCA
s100a7	CTTGTCCCTGGAGGAGTTGA	GCTTGCCCAAGATGTACAGG
s100a8	GGAAATCACCATGCCCTCTA	TGGCTGTCTTTGTGAGATGC
s100a9	TCATCGACACCTTCCATCAA	TCAACTTTGCCATCAGCATC
Ccl5	CCCTCACCATCATCCTCACT	CTGCAAGATTGGAGCACTTG
Ccl8	ACGCTAGCCTTCACTCCAAA	GAAGGGGGATCTTCAGCTTT
Ccl17 (Tarc)	AGTGGAGTGTTCCAGGGATG	CTGGTCACAGGCCGTTTTAT
Ccl20	CGACTGTTGCCTCTCGTACA	AGCCCTTTTCACCCAGTTCT
Ccl22 (Mdc)	TTCTTGCTGTGGCAATTCAG	GCAGGATTTTGAGGTCCAGA
Cxcl1	GCCTATCGCCAATGAGCTG	AAGGGAGCTTCAGGGTCAAG
Cxcl9	GATAAGGAATGCACGATGCTC	AGTCCGGATCTAGGCAGGTT
Cxcl10	GCTGCAACTGCATCCATATC	TTTTGGCTAAACGCTTTCATT
Cxcl11	GGGATGAAAGCCGTCAAAA	CTGCATTATGAGGCGAGCTT
Klk7	CTCCACAAAGACCCACGTCA	GTTTTCCCCAGCAGGTCCTT
Klk14	ATGTTCCTTCTACTGATCATA	AGCAGCAGTGATGACCCATTGATC
Klk5 real-time PCR	GTGCAGCGGTCATGGCGAGGACCG	AATCCGTGCTGAGGTCTCTGTTG
Klk5 sqPCR	GTGCAGCGGTCATGGCGAGGACCG	GGTGTGGCTCATTAGTTGGAGTTC
Klk5 wt allele	GCAGCTAGAGTTAAGAGCTC	GGTCAGAACTGTGTAGGC
Klk5 ko allele	GCAGCTAGAGTTAAGAGCTC	GACCACCTCATCAGAAGCAG
Spink5 wt allele	GTTCTCAAGGAGTCTAACAT	CTTGTGTGAGATAAAATGCC
Spink5 ko allele	GTTCTCAAGGAGTCTAACAT	ATGCGAAGTGGACCTGGGAC

Table S1. Primer sequences and conditions used for genotyping, real-time PCR, and semi-quantitative RT-PCR.

*sq: semi-quantitative PCR
